# Stigma receptors control intraspecies and interspecies barriers in Brassicaceae

**DOI:** 10.1038/s41586-022-05640-x

**Published:** 2023-01-25

**Authors:** Jiabao Huang, Lin Yang, Liu Yang, Xiaoyu Wu, Xiaoshuang Cui, Lili Zhang, Jiyun Hui, Yumei Zhao, Hongmin Yang, Shangjia Liu, Quanling Xu, Maoxuan Pang, Xinping Guo, Yunyun Cao, Yu Chen, Xinru Ren, Jinzhi Lv, Jianqiang Yu, Junyi Ding, Gang Xu, Nian Wang, Xiaochun Wei, Qinghui Lin, Yuxiang Yuan, Xiaowei Zhang, Chaozhi Ma, Cheng Dai, Pengwei Wang, Yongchao Wang, Fei Cheng, Weiqing Zeng, Ravishankar Palanivelu, Hen-Ming Wu, Xiansheng Zhang, Alice Y. Cheung, Qiaohong Duan

**Affiliations:** 1 State Key Laboratory of Crop Biology, Shandong Agricultural University, Tai’an, China; 2 College of Horticulture Science and Engineering, Shandong Agricultural University, Tai’an, China; 3 Institute of Horticulture, Henan Academy of Agricultural Sciences, Zhengzhou, China; 4 Computer Network Information Centre, Chinese Academy of Sciences, Beijing, China; 5 National Key Laboratory of Crop Genetic Improvement, Huazhong Agricultural University, Wuhan, China; 6 Shandong Yiyi Agricultural Science and Technology Co., Ltd, Tai’an, China; 7 College of Horticulture, Qingdao Agricultural University, Qingdao, China; 8 International Flavors & Fragrances, Wilmington, DE USA; 9 School of Plant Sciences, University of Arizona, Tucson, AZ USA; 10 Department of Biochemistry and Molecular Biology, Molecular Cell Biology and Plant Biology Programs, University of Massachusetts, Amherst, MA USA

**Keywords:** Plant signalling, Fertilization

## Abstract

Flowering plants have evolved numerous intraspecific and interspecific prezygotic reproductive barriers to prevent production of unfavourable offspring^1^. Within a species, self-incompatibility (SI) is a widely utilized mechanism that rejects self-pollen^2,3^ to avoid inbreeding depression. Interspecific barriers restrain breeding between species and often follow the SI × self-compatible (SC) rule, that is, interspecific pollen is unilaterally incompatible (UI) on SI pistils but unilaterally compatible (UC) on SC pistils^1,4–6^. The molecular mechanisms underlying SI, UI, SC and UC and their interconnections in the Brassicaceae remain unclear. Here we demonstrate that the SI pollen determinant *S*-locus cysteine-rich protein/*S*-locus protein 11 (SCR/SP11)^2,3^ or a signal from UI pollen binds to the SI female determinant *S*-locus receptor kinase (SRK)^2,3^, recruits FERONIA (FER)^7–9^ and activates FER-mediated reactive oxygen species production in SI stigmas^10,11^ to reject incompatible pollen. For compatible responses, diverged pollen coat protein B-class^12–14^ from SC and UC pollen differentially trigger nitric oxide, nitrosate FER to suppress reactive oxygen species in SC stigmas to facilitate pollen growth in an intraspecies-preferential manner, maintaining species integrity. Our results show that SRK and FER integrate mechanisms underlying intraspecific and interspecific barriers and offer paths to achieve distant breeding in Brassicaceae crops.

## Abstract (web summary)

A signalling mechanism ensuring intraspecies and interspecies reproductive barriers in flowering plants is uncovered.

## Main

In flowering plants, prezygotic reproductive barriers may occur at one or more check points before fertilization to prevent production of unfavourable offspring^1^. In nature, the stigma of an open flower is exposed to pollen from its own species, closely and distantly related interspecies, therefore must respond accordingly. Within a species, SI is widely utilized as a mechanism to avoid inbreeding depression and promote hybrid vigour by rejecting self*-*pollen and accepting intraspecific cross-compatible (CP) pollen^2,3^. Between species, interspecific incompatibility maintains species integrity and often follows the SI × SC rule, that is, interspecific pollen is UI on SI pistils but UC on SC pistils^1,4–6^. So far, little is known about the molecular mechanisms regulating these compatibility systems and their interconnections in the Brassicaceae.

In Brassicaceae SI, self-pollen is recognized by the ligand–receptor interaction between the pollen-expressed SCR/SP11 and its receptor, the stigma-expressed, plasma membrane-localized SRK^2,3^. This activates two downstream positive regulators of SI, the *M*-locus protein kinase (MLPK)^15^ and ARM-repeat containing 1 (ARC1) E3 ubiquitin ligase^16^. How MLPK functions remains unclear^2,15,17^. ARC1 targets compatible factors such as the exocyst component EXO70A1 for degradation, blocking pollen hydration^2,16,18^. We discovered that the female fertility regulator FER receptor kinase^7–9^ maintains a stigmatic gate in *Arabidopsis thaliana*^14^ and has a dual role in rejecting SI pollen and facilitating SC pollen germination in *Brassica rapa*^10,11^ by signalling a rapid elevation of stigmatic reactive oxygen species (ROS) or its decline, respectively.

## SRK controls rejection of UI pollen

**Fig. 1:**
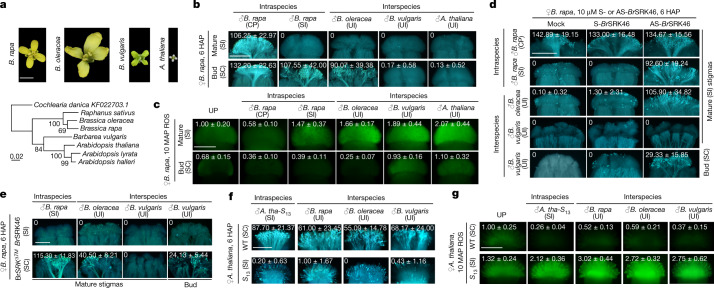
SRK controls stigmatic ROS in SI to reject intraspecific and interspecific pollen. **a**, Open flowers and phylogenetic tree for Brassicaceae species from maximum likelihood analysis using internal transcribed spacer sequences. Distantly related *Cochlearia danica* served as an outgroup. Scale bar, mean number of nucleotide substitutions per site. **b**, Aniline blue staining showing intraspecific and interspecific pollen growth in mature and bud-stage *B. rapa* stigmas. **c**, H_2_DCFDA staining of ROS in unpollinated (UP) or pollinated *B. rapa* stigmas. **d**,**e**, Relaxed SI and UI in AS-*Br*SRK46-treated *B. rapa S*_46_ stigmas (**d**) and stigmas from SRK-defective *B. rapa*, Br*SRK*^*∆TM*^ (**e**). **f**,**g**, Intraspecific and interspecific pollen (**f**) and ROS responses (**g**) in stigmas from SC *A. thaliana*, that is, wild type (WT), and stigmas from SI *A. thaliana* expressing *A. halleri S*_13_ genes (*A. tha*-*S*_13_). The values in the **b**–**g** images, shown as average ± s.d., indicate average number of pollen tubes in the stigma (**b**,**d**,**e**,**f**) and average ROS intensity (**c**,**g**). The same data are also presented in box plots with all data points (Extended Data Figs. 1, 2). Scale bars, 0.5 cm (**a**), 500 μm (**b**–**e**) and 200 μm (**f**,**g**). Each experiment was repeated at least three times with consistent results.

We first characterized pollen-induced responses during SI, SC, UI or UC pollination in *B. rapa* and *A. thaliana* stigmas by depositing pollen from *B. rapa*, closely related interspecific *Brassica oleracea*, more distant intergeneric *Barbarea vulgaris* and *A. thaliana*^19,20^ (Fig. 1a). Of particular interest is crosses involving *B. vulgaris* owing to its high resistance to fungal and insect pathogens^21,22^, but intergeneric barriers prevent introgression of its desirable traits into *Brassica* crops.

**Extended Data Fig. 1:**
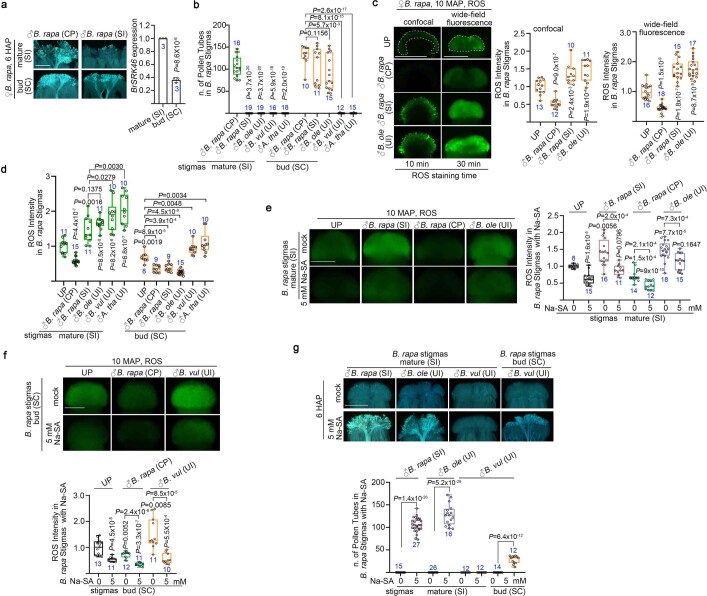
ROS underlies the rejection of interspecific pollen during UI. **a**, The developmentally regulated SI and the progressive increase in the amount of SRK from bud stage to maturity. **b**, The number of intra*–* and various interspecific pollen tubes in mature or bud-stage *B. rapa* stigmas. Mature *B. rapa* stigmas rejected SI and all the interspecific pollen examined, but bud-stage *B. rapa* stigmas allowed the growth of SI pollen and interspecific *B. oleracea* pollen, and still rejected intergeneric *B. vulgaris* and *A. thaliana* pollen. See Fig. 1b. **c**, Imaging of H_2_DCFDA-stained ROS under confocal microscope and under wide-field fluorescence microscope showed comparable changes of ROS in *B. rapa* stigmas Dotted line outlined the area for ROS quantification. Wide-field observation, with its considerably high expedition to sample entire stigma specimens, was used for the massive amount of data gathering required for this study. **d**, ROS intensity in unpollinated (UP) or pollinated *B. rapa* stigma. See Fig. 1c. **e**–**g**, Scavenging ROS by Na-SA suppressed stigmatic ROS induction, and promoted the growth of SI pollen and *B. oleracea* pollen in mature and *B. vulgaris* pollen in bud-stage *B. rapa* stigmas. Scale bars, 500 μm. Data bar (**a**): average ± SD. Average relative expression levels from three biological replicates of stigmas (two tailed *t*-test, n = 3). Box plots (**b**–**g**): centre line, median; box limits, lower and upper quartiles; dots, individual data points; whiskers, highest and lowest data points. n (in blue), number of stigmas. *P* values, two-tailed *t*-tests. Each experiment was repeated at least thrice with consistent results. Source data

We used *B. rapa* var. *pekinensis*, an economically important vegetable crop also called heading Chinese cabbage, as a representative SI species for pollen-induced responses on the stigma. A hallmark of SI in Brassicaceae is that its strength is developmentally regulated, due to the progressive increase in the amount of SRK accumulation from bud stage to maturity^23^ (Extended Data Fig. 1a). Compared with extensive CP pollen tube penetration into mature *B. rapa* stigmas, SI and interspecific pollen were severely inhibited (Fig. 1b and Extended Data Fig. 1b). Bud-stage *B. rapa* stigmas were comparably well penetrated by both SI and *B. oleracea* pollen, reflecting the involvement of an SRK-mediated mechanism in UI, mimicking SI. However, they remained impenetrable by *B. vulgaris* and *A. thaliana* pollen (Fig. 1b and Extended Data Fig. 1b), and it is likely that even the low level of SRK in bud-stage *B. rapa* stigmas is enough to reject intergeneric pollen.

Given that the profile of SRK expression aligns with that of ROS accumulation^10^, we examined whether stigmatic redox status has a crucial role in the UI response. Like the SI-induced stigmatic ROS increase^10,11^ but contrary to CP-induced ROS decline, UI pollen stimulated notable ROS increase in mature *B. rapa* stigmas. Bud-stage stigma ROS was reduced by *B. oleracea* pollen but was increased by *B. vulgaris* and *A. thaliana* pollen by 10 min after pollination (Fig. 1c and Extended Data Fig. 1c,d). Furthermore, sequestrating ROS by sodium salicylate (Na-SA)^8,24,25^ not only broke the barrier for *B. oleracea* pollen on mature *B. rapa* stigmas but also the barrier for *B. vulgaris* pollen on bud-stage stigmas (Extended Data Fig. 1e–g). Together, these results support UI pollen-induced ROS being essential for their rejection on SI stigmas.

**Extended Data Fig. 2:**
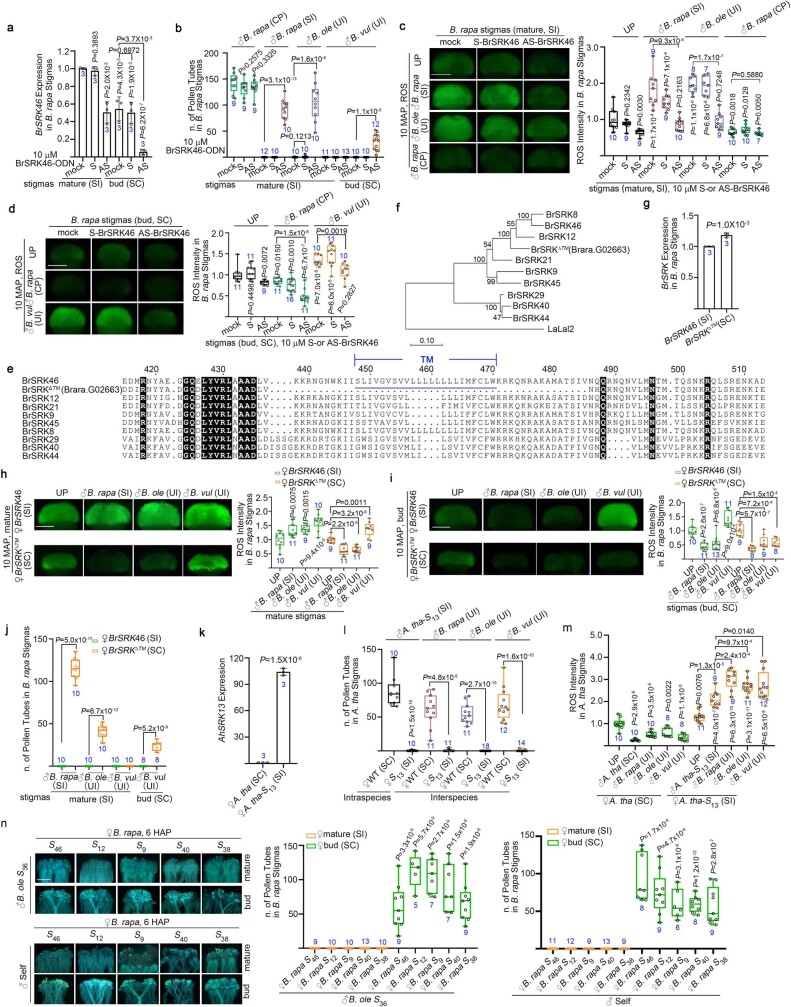
SRK underlies the rejection of interspecific pollen during UI. **a**, Quantitative RT-PCR showing AS-BrSRK46 treatment reduced *BrSRK46* expression in mature *B. rapa* stigmas (*S*_46_) and further reduced its already low level in bud-stage stigmas (*S*_46_). **b**, AS-BrSRK46 treatment of mature *B. rapa* stigmas (*S*_46_) broke the inhibition to *B. oleracea* pollen not *B. vulgaris* pollen. AS-BrSRK46 treatment of bud-stage stigmas (*S*_46_) broke inhibition to *B. vulgaris* pollen. See Fig. 1d. **c**, **d**, AS-BrSRK46 treatment reduced ROS in mature *B. rapa* stigmas (*S*_46_) and further reduced its already low ROS in bud-stage stigmas (*S*_46_). **e**–**g**, Sequence alignment (**e**), phylogenetic analysis (**f**) and expression of SRK in FPsc (Brara.G02663) (**g**). SRK from FPsc lacks the coding sequence for the transmembrane domain and we name it *BrSRK*^*∆TM*^ hereafter. **h**–**j**, Mature and bud-stage stigmas of FPsc were defective in SI and UI pollen-triggered ROS increase **(h**, **i**) and the rejection of SI and UI pollen (**j**). See Fig. 1e. **k**, *SRK13* expression from SI *A. thaliana* stigmas transformed with *Arabidopsis halleri* AhSP11/SCR13-AhSRK13-AhARC1. These three transgenes were in a single construct. **l**, **m**, The number of intra*–* or interspecific pollen tubes (**l**) and ROS changes (**m**) in SC *A. thaliana* stigmas and in SI *A. thaliana* stigmas. See Fig. 1f, g. **n**, *B. oleracea* pollen of *S*_36_-haplotype was rejected in *B. rapa* stigmas of *S*_46_, *S*_12_, *S*_9_, *S*_40_, or *S*_38_, showing the dependence on *S*RK but the independence of SCR-SRK interaction in the rejection of interspecific pollen. Scale bars, 500 μm (**c**, **d**, **h**, **i**, **n**). Data bars (**a**, **g**, **k**): average ± SD. Average relative expression levels from three biological replicates of stigmas (two tailed *t*-test, n = 3). Box plots (**b**–**d**, **h**–**j**, **l**–**n**): centre line, median; box limits, lower and upper quartiles; dots, individual data points; whiskers, highest and lowest data points. n (in blue), number of stigmas. *P* values, two-tailed *t*-tests. Each experiment was repeated at least thrice with consistent results. Source data

Next, we tested whether SRK, in addition to rejecting SI pollen, also underlies the UI response. Treating *S*_46_ *B. rapa* with AS-*Br*SRK46 (antisense oligodeoxyribonucleotide (AS-ODN) that specifically suppresses *Br*SRK46)^10^, allowed the penetration of *B. oleracea* pollen tubes into mature stigmas and *B. vulgaris* pollen tubes into bud-stage stigmas, consistent with ROS reduction (Fig. 1d and Extended Data Fig. 2a–d). Moreover, ‘*B. rapa* fast plant self-compatible (FPsc)’ expresses a transmembrane domain-deleted SRK, hereafter named Br*SRK*^*ΔTM*^, failed to respond with SI and UI pollen-triggered ROS increase and showed compromised rejection of SI and UI pollen (Fig. 1e and Extended Data Fig. 2e–j). Together, these results clearly demonstrate that SRK not only controls SI but also the rejection of interspecific and intergeneric pollen.

Furthermore, *A. thaliana*, a typical SC Brassicaceae with loss-of-function mutations or complete deletions of the *SRK* and/or *SCR* genes^23,26,27^, allowed pollen penetration from *A. thaliana*, *B. rapa*, *B. oleracea* and *B. vulgaris*, and showed stigmatic ROS reduction after pollination. However, transgenic *A. thaliana* expressing SP11/SCR, SRK and ARC1 from the *A. halleri* of *S*_13_ (referred to as *A. tha*-*S*_13_ hereafter), which recapitulates SI^28^, strongly inhibited interspecific pollen and rapidly increased stigmatic ROS (Fig. 1f,g and Extended Data Fig. 2k–m). In addition, *B. oleracea S*_36_ pollen was similarly rejected by *B. rapa* stigmas of several different *S*-haplotypes (Extended Data Fig. 2n), suggesting that SRK variations do not differ in their function in UI. Together, these results demonstrated unambiguously that SRK and SRK-dependent high stigmatic ROS levels positively correlate with the rejection of UI pollen, similar to the SI response.

## UI and SI activate SRK–FER-regulated ROS

**Fig. 2:**
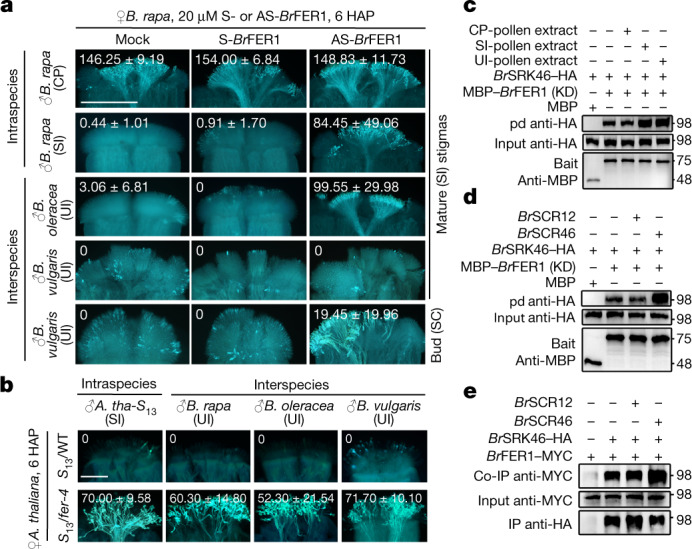
UI and SI converge on stigmatic ROS activation for pollen rejection via the SRK*–*FER interaction. **a**,**b**, Treating *B. rapa* stigmas with AS-*Br*FER1 or crossing *fer-4* into SI *A. thaliana* stigmas, *S*_13_/*fer-4*, relaxed SI and UI. The values in the **a**,**b** images, shown as average ± s.d., indicate average number of pollen tubes in the stigma. The same data are also presented in box plots with all data points (Extended Data Fig. 3). **c**–**e**, Pull-down (pd) (**c**,**d**) and co-immunoprecipitation (co-IP) (**e**) assays showing protein extracts from SI and UI pollen. *Br*SCR46 augmented the *Br*SRK46–*Br*FER1 interaction. The protein samples were derived from the same experiment and the blots were processed in parallel (**c**–**e**). For gel source data, see Supplementary Fig. 1. Scale bars, 500 μm (**a**) and 200 μm (**b**). Each experiment was repeated at least three times with consistent results.

**Extended Data Fig. 3:**
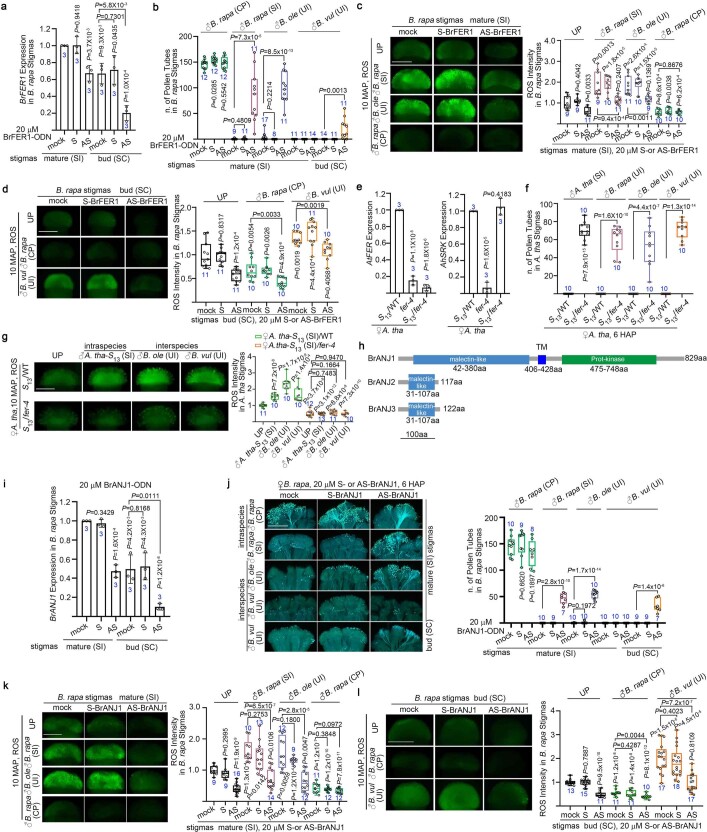
FER/ANJ signalling regulates stigmatic ROS during UI. **a**, **b**, Quantitative RT-PCR showing AS-BrFER1 treatment suppressed the expression of *BrFER1*(**a**) and promoted the growth of SI pollen and *B. oleracea* pollen in mature *B. rapa* stigmas and *B. vulgaris* pollen in bud stage (**b**) *B. rapa* stigmas. See Fig. 2a. **c**, **d**, AS-BrFER1 treatment suppressed SI and UI-induced ROS increase in mature and bud stage *B. rapa* stigmas at 10 MAP. **e**, **f**, The expression of *FER* and *SRK* in stigmas of *A. tha* (SI)*/fer-4* (**e**) and loss of FER in SI *A. thaliana* promoted the growth of SI pollen, *B. oleracea* pollen and *B. vulgaris* pollen in SI *A. thaliana* stigmas (**f**). See Fig. 2b. **g**, Loss of FER in SI *A. thaliana* suppressed SI and UI-induced ROS increase in *A. tha* (SI)*/fer-4* stigmas at 10 MAP. **h**, Domain structures of BrANJs proteins showing only BrANJ1 have intact extracellular and intracellular domain. **i**, **j**, AS-BrANJ1 treatment suppressed the expression of *BrANJ1*(**i**) and promoted the growth of SI pollen and *B. oleracea* pollen in mature *B. rapa* stigmas and *B. vulgaris* pollen in bud stage *B. rapa* stigmas (**j**). **k**, **l**, AS-BrANJ1 treatment suppressed SI and UI-induced ROS increase in mature (**k**) and bud stage (**l**) *B. rapa* stigmas at 10 MAP. Scale bars, Scale bars, 500 μm (**c**, **d**, **j**, **k**, **l**), 200 μm (**g**). Data bars (**a**, **e**, **i**): average ± SD. Average relative expression levels from three biological replicates of stigmas (two tailed *t*-test, n = 3). Box plots (**b**–**d**, **f**, **g**, **j**–**l**): centre line, median; box limits, lower and upper quartiles; dots, individual data points; whiskers, highest and lowest data points. n (in blue), number of stigmas. *P* values, two-tailed *t*-tests. Each experiment was repeated at least thrice with consistent results. Source data

**Extended Data Fig. 4:**
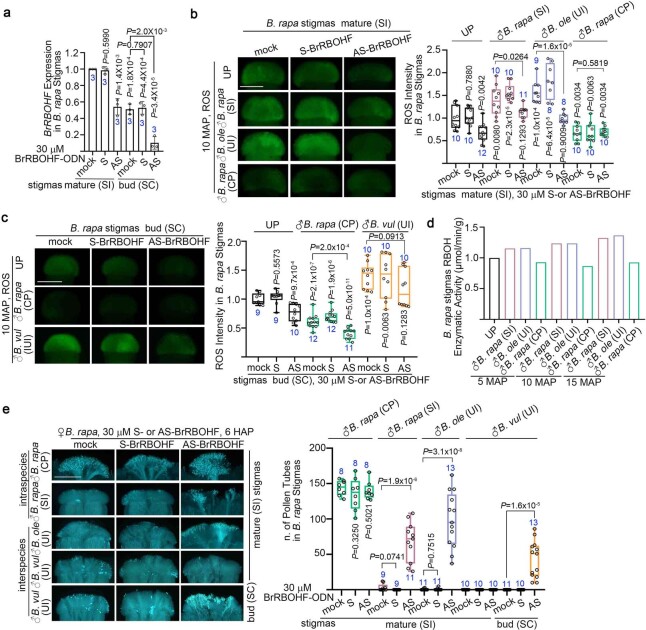
ROS are produced by RBOHs during UI. **a**, Quantitative RT-PCR showing AS-BrRBOHF treatment suppressed the expression of *BrRBOHF*. **b**, **c**, AS-BrRBOHF treatment suppressed SI and UI-induced ROS increase in mature (**b**) and bud-stage (**c**) *B. rapa* stigmas at 10 MAP. **d**, The RBOH enzymatic activity in mature *B. rapa* stigmas were increased after pollination with SI and UI pollen but decreased after pollination with CP pollen. **e**, AS-BrRBOHF treatment promoted the growth of SI pollen and *B. oleracea* pollen in mature and *B. vulgaris* pollen in bud-stage *B. rapa* stigmas. Scale bars, 500 μm. Data bar (**a**): average ± SD. Average relative expression levels from three biological replicates of stigmas (two tailed *t*-test, n = 3). Data bars (**d**): Average activity of ROS producing enzymes from three technical replicates of one stigma sample containing 100 stigmas (two tailed *t*-test, n = 3). Box plots (**b**, **c**, **e**): centre line, median; box limits, lower and upper quartiles; dots, individual data points; whiskers, highest and lowest data points. n (in blue), number of stigmas. *P* values, two-tailed *t*-tests. Each experiment was repeated at least thrice with consistent results unless otherwise specified. Source data

**Extended Data Fig. 5:**
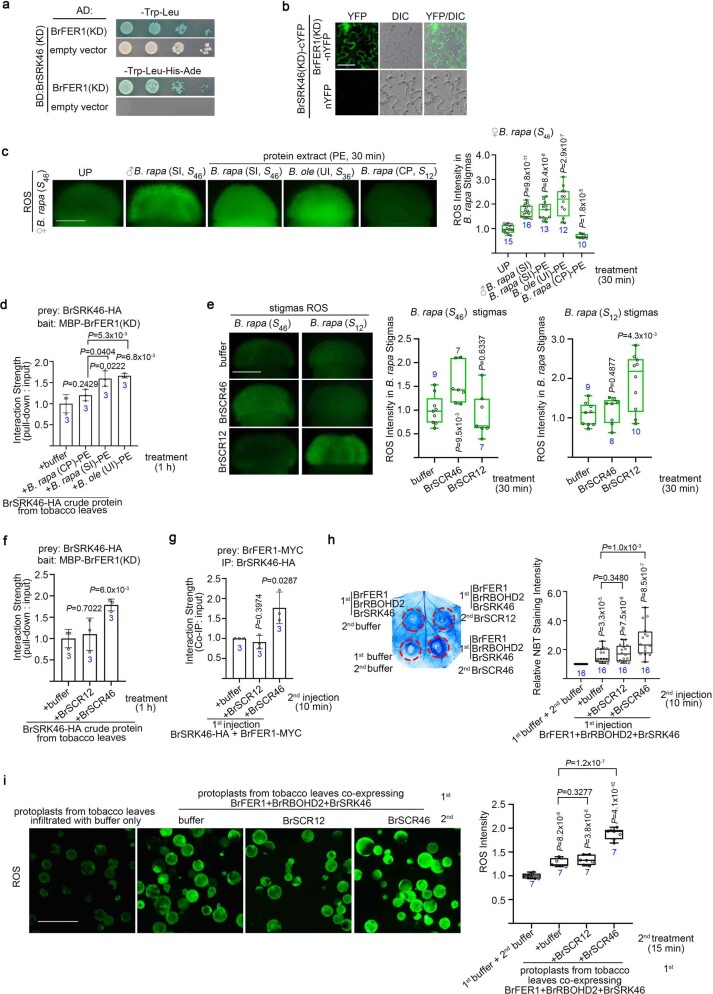
SRK is correlated with stigmatic ROS during UI. **a**, **b**, BrSRK46 kinase domain (KD) interacts with BrFER1 (KD) in yeast two-hybrid assay (**a**) and BiFC (**b**). **c**, **d**, Protein extract from SI pollen and *B. oleracea* pollen (*S*_36_), but not that from CP pollen (*S*_12_), increased ROS in *B. rapa* stigmas (*S*_46_) (**c**), and enhanced interaction between BrSRK46 and BrFER1 (**d**) in the pull-down assay. See Fig. 2c. **e**–**g**. ROS in *B. rapa* stigmas (*S*_46_) were increased (**e**), and the BrSRK46-BrFER1 interaction enhanced in the pull-down (**f**) and Co-IP assays (**g**), by GST-BrSCR46, not GST-BrSCR12, which is active for *S*_12_ stigma. The protein samples were derived from the same experiment and the blots were processed in parallel (**d**, **f**, **g**). For gel source data, see Supplementary Fig. 1. See Fig. 2d, e. **h**, **i**, Nitro blue tetrazolium staining or tobacco leaves and H_2_DCFDA staining of protoplasts from tobacco leaves co-expressing BrFER1, BrRBOHD2, and BrSRK46 showing the enhancement of ROS by BrSCR46, not by BrSCR12. Scale bars, 500 μm (**c**, **e**); 50 μm (**i**). Data bars (**d**, **f**, **g**): average ± SD. Average relative intensities from three biological replicates of the blots shown in Fig. 2c–e (two tailed *t*-test, n = 3). Box plots (**c**, **e**, **h**, **i**): centre line, median; box limits, lower and upper quartiles; dots, individual data points; whiskers, highest and lowest data points. n (in blue), number of stigmas, tobacco leaves, protoplast samples. *P* values, two-tailed *t*-tests. Each experiment was repeated at least thrice with consistent results. Source data

Given the role of FER in regulating ROS^8,29^, we suppressed Br*FER1* in *B. rapa* stigmas by AS-ODN or crossed *fer-4* mutant into SI *Arabidopsis*, and observed effective inhibition of SI and UI pollen-triggered stigmatic ROS increase, and marked SI and UI breakdown (Fig. 2a,b and Extended Data Fig. 3a–g). Suppressing *Br*ANJEA1 (*Br*ANJ1), which complexes with FER^14^, or *Br*RBOHF, which produces ROS^10^, also severely inhibited ROS increase and compromised SI and UI pollen rejection (Extended Data Figs. 3h–l and 4). Furthermore, yeast two-hybrid, bimolecular fluorescent complementation (Extended Data Fig. 5a,b) and protein pull-down assays demonstrated that *Br*SRK46 interacted with *Br*FER1 (Fig. 2c,d). Furthermore, protein extracts from SI and UI pollen, and SI determinant *Br*SCR46, markedly augmented *Br*SRK46–HA pulled down by MBP–*Br*FER1 (kinase domain (KD)), whereas CP pollen extracts and *Br*SCR12 had no effect (Fig. 2c,d and Extended Data Fig. 5c–f). Moreover, when co-expressed in tobacco leaves, *Br*SCR46 enhanced *Br*FER1–MYC co-immunoprecipitation with *Br*SRK46–HA and ROS production in tobacco leaves (Fig. 2e and Extended Data Fig. 5g–i). Together, these results suggest that SCR from SI pollen^2,3^ and an unknown signal from UI pollen trigger SRK-dependent activation of FER-regulated ROS production by boosting SRK–FER interaction to reject the incompatible pollen.

**Extended Data Fig. 6:**
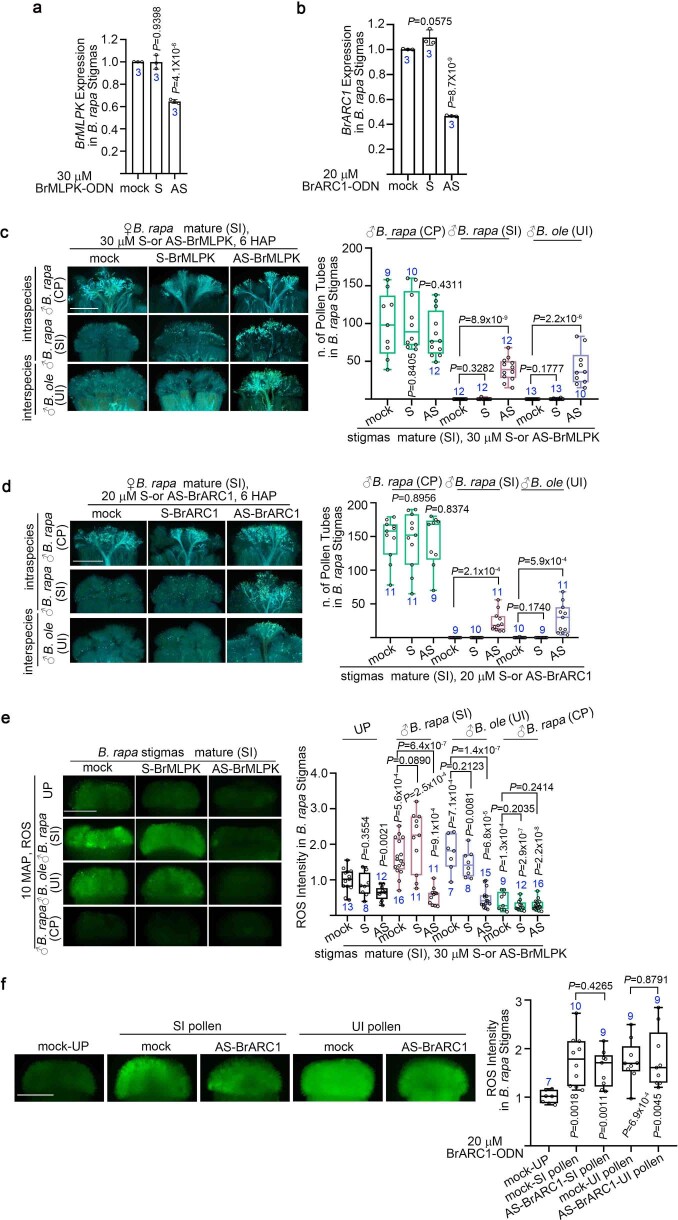
The relationship of FER-ROS signaling with MLPK and ARC1 during SI and UI. **a**, **b**, Quantitative RT-PCR showing AS-BrMLPK and AS-BrARC1 treatment suppressed the expression of *BrMLPK* and *BrARC1*, respectively, in mature *B. rapa* stigmas. **c**, **d**, AS-BrMLPK (**c**) and AS-BrARC1 (**d**) treatment promoted the growth of SI and UI pollen tubes in mature *B. rapa* stigmas (*S*_46_). **e**, AS-BrMLPK treatment of mature *B. rapa* stigmas (*S*_46_) inhibited SI and UI pollen-induced ROS increase. **f**, AS-BrARC1 treatment of mature *B. rapa* stigmas (*S*_46_) did not affect SI and UI pollen-induced ROS increase. Scale bars, 500 μm. Data bars (**a**, **b**): average ± SD. Average relative expression levels from three biological replicates of stigmas (two tailed *t*-test, n = 3). Data bar: average ± s.d. Box plots (**c**–**f**): centre line, median; box limits, lower and upper quartiles; dots, individual data points; whiskers, highest and lowest data points. n (in blue), number of stigmas. *P* values, two-tailed *t*-tests. Each experiment was repeated at least thrice with consistent results. Source data

We then explored how the FER–ROS signalling pathway might connect with known SRK signalling^15–18^. Suppressing *Br*MLPK and *Br*ARC1 by AS-ODN allowed the penetration of SI as well as *B. oleracea* pollen tubes in mature *B. rapa* stigmas (Extended Data Fig. 6a–d). However, although AS-*Br*MLPK inhibited SI and UI-triggered ROS increase, AS-*Br*ARC1 did not (Extended Data Fig. 6e,f). Together, these results suggest that after SRK senses SI and UI pollen, it activates two parallel intracellular signalling pathways: the FER to ROS pathway that mediates pollen rejection, possibly involving MLPK, and the ARC1-mediated pathway for the degradation of compatible factors required for pollen growth.

## FER and PCP-Bs favour SC over UC pollen

**Fig. 3:**
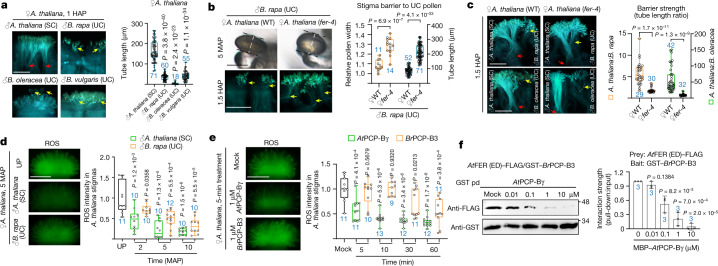
Species-preferential interaction between PCP-Bs and FER maintains interspecies barrier. **a**, In *A. thaliana* stigmas, *A. thaliana* pollen tubes were longer than those of *B. rapa*, *B. oleracea* and *B. vulgaris* at 1 HAP. **b**, Faster hydration and growth of *B. rapa* pollen on *A. thaliana fer-4* stigmas. The orange plots indicate relative pollen width and the blue plots indicate pollen tube length. The equatorial diameter of a pollen grain, indicated by white dashed lines, was measured in ImageJ for pollen width. **c**, Relaxed interspecies barrier in *fer-4* stigmas. Ratios of pollen tube length in intraspecies and interspecific crosses are used as a measure for barrier strength. In **a**–**c**, the arrows indicate pollen tube front. **d**,**e**, Species-preferential ROS reduction in *A. thaliana* stigmas by pollen (**d**) and PCP-Bs (**e**) from intraspecies and interspecies. **f**, Pull-down assay showing *At*PCP-Bγ competed dose-dependently with GST–*Br*PCP-B3 for interaction with *At*FER (ED)–FLAG. The protein samples were derived from the same experiment and the blots were processed in parallel (**f**). For gel source data, see Supplementary Fig. 1. Scale bars, 200 μm (**a**–**e**) and 20 μm (pollen in **b**). For box plots (**a**–**e**), the centre line indicates the median, the box limits denote the lower and upper quartiles, the dots indicate individual data points, and the whiskers denote the highest and lowest data points. *P* values were determined by two-tailed Student *t*-tests. *n* (in blue) indicates the number of stigmas or pollen grains. In **f**, for the data bar, average ± s.d. is shown; average intensities from three biological replicates of the blot are represented on the left (two-tailed *t*-test, *n* = 3). Each experiment was repeated at least three times with consistent results. Source data

**Extended Data Fig. 7:**
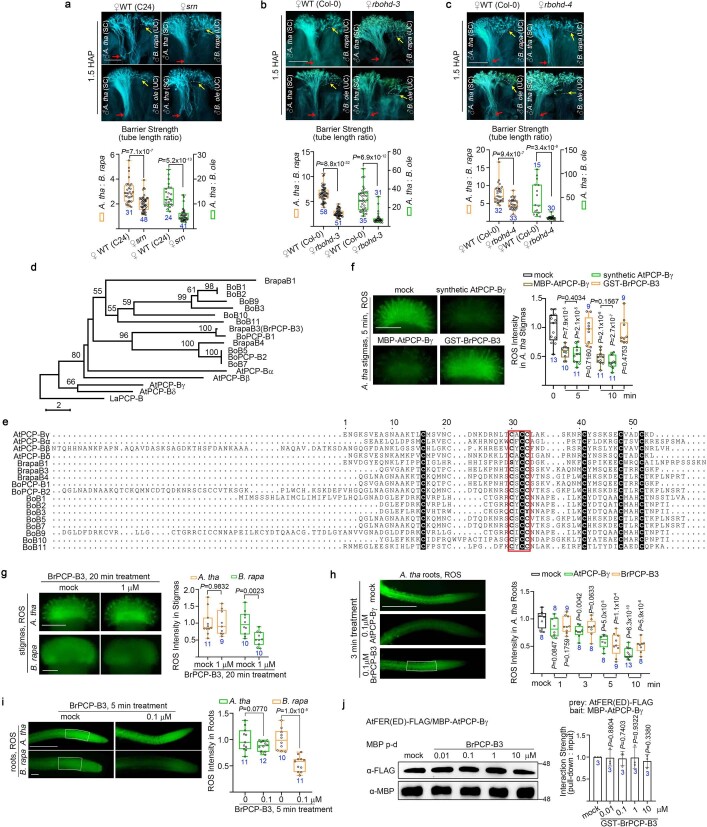
Species-specific match of PCP-Bs and FER. **a**–**c**, When observed at 1.5 HAP, the reduced reproductive barrier to *B. rapa* and *B. oleracea* pollen in *A. thaliana fer* mutant (**a**, *srn*) and *rbohd* mutant (**b**, **c**) stigmas was obvious. See also Fig. 3c. **d**, Phylogenetic analysis of PCP-B genes from *A. thaliana*, *B. oleracea*, and *B. rapa*. We name BrapaB3^12^, an ortholog of AtPCP-Bγ, BrPCP-B3 hereafter. **e**, Amino acid sequence alignment of PCP-Bs from *A. thaliana*, *B. oleracea*, and *B. rapa* shows the comparison of PCP-B sequences. They all shared a common pattern of seven or eight cysteines in the mature polypeptide. Red rectangle shows the conserved cysteines (C30, C32, C33 of AtPCP-B), mutation of which render a loss in the ability for stigmatic ROS reduction and pollen hydration^14^. **f**, Synthetic AtPCP-Bγ and recombinant MBP-AtPCP-Bγ show similar function in reducing ROS of UP *A. thaliana* stigmas, but GST-BrPCP-B3 shows no effect in reducing ROS at 5 or 10 MAT. **g**, *B. rapa* stigmas and *A. thaliana* stigmas, which express divergent FER, responded species-preferentially to BrPCP-B3 in reducing ROS. **h**, AtPCP-Bγ was much faster than GST-BrPCP-B3 in reducing ROS of *A. thaliana* roots, which also express FER^28^. **i**, GST-BrPCP-B3 was more effective in reducing ROS of *B. rapa* roots than that of *A. thaliana* roots. **j**, Quantified data of pull-down assay showing the inefficiency of GST-BrPCP-B3 to compete with AtPCP-Bγ in interaction with AtFER (ED)-FLAG. The protein samples were derived from the same experiment and the blots were processed in parallel. For gel source data, see Supplementary Fig.1. See also Fig. 3f. Scale bars, 200 μm (**a**–**c**, **f**, **g**) and roots (**h**, **i**). Data bar (**j**): average ± SD. Average relative intensities from three biological replicates of the blot represented on the left (two tailed *t*-test, n = 3). Box plots (**a**–**c**, **f**, **g**–**i**): centre line, median; box limits, lower and upper quartiles; dots, individual data points; whiskers, highest and lowest data points. n (in blue), number of stigmas or roots. *P* values, two-tailed *t*-tests. Each experiment was repeated at least thrice with consistent results. Source data

We used *A. thaliana* for pollen-induced responses on SC stigmas. Although pollen tubes from *B. rapa*, *B. oleracea* or *B. vulgaris* all penetrated *A. thaliana* stigmas by 6 h after pollination (HAP) (Fig. 1f), UC pollen tubes were much shorter than SC pollen tubes when examined earlier (1 HAP) (Fig. 3a), suggesting a more stringent barrier for UC pollen. We determined that FER has an important role in suppressing UC pollen in *A. thaliana* stigma as *B. rapa* pollen hydrated considerably faster and their tubes were much longer in *fer-4* stigmas than those in wild type by 1.5 HAP (Fig. 3b). Moreover, simultaneous deposition of *A. thaliana* with *B. rapa* pollen, or *A. thaliana* with *B. oleracea* pollen on the same *A. thaliana* stigma showed a notable reduction in stigmatic barrier strength in *fer* and *rbohd* stigmas (Fig. 3c and Extended Data Fig. 7a–c). UC pollen was also slower than SC pollen in inducing ROS decline (Fig. 3d), consistent with FER-mediated ROS functioning as a barrier in SC stigmas to discriminate against interspecific pollen for species integrity.

Pollen coat proteins B-class (PCP-Bs) are highly polymorphic cysteine-rich peptides that are widespread in the Brassicaceae (Extended Data Fig. 7d,e) and are important for pollen hydration^12–14^. In *A. thaliana*, PCP-Bγ binds to FER and reduces stigmatic ROS to allow pollen growth^14^. We therefore examined the efficacy of PCP-Bs from different species in suppressing ROS. On *A. thaliana* stigmas, *At*PCP-Bγ induced a marked reduction of ROS as early as 5 min after treatment, but *Br*PCP-B3 (ref. ^12^) reduced ROS to comparable levels only by 60 min after treatment. Reciprocally, *B. rapa* stigma ROS responded to *Br*PCP-B3 earlier than *A. thaliana* stigmas (Fig. 3e and Extended Data Fig. 7f,g). Moreover, species-specific PCP-B-induced ROS suppression was recapitulated in *Arabidopsis* roots, which also express FER^29^ (Extended Data Fig. 7h,i), thus providing further support. Pull-down assays showed that *At*PCP-Bγ competed dose-dependently with *Br*PCP-B3, but not vice versa, for interaction with the *At*FER extracellular domain (ED) (Fig. 3f and Extended Data Fig. 7j). Together, these results suggest that the species-specific match between PCP-Bs and FER is important for a rapid compatibility response and serves as an interspecific barrier to favour intraspecific pollen for fertilization.

## Compatible pollen induced-NO reduces ROS

**Fig. 4:**
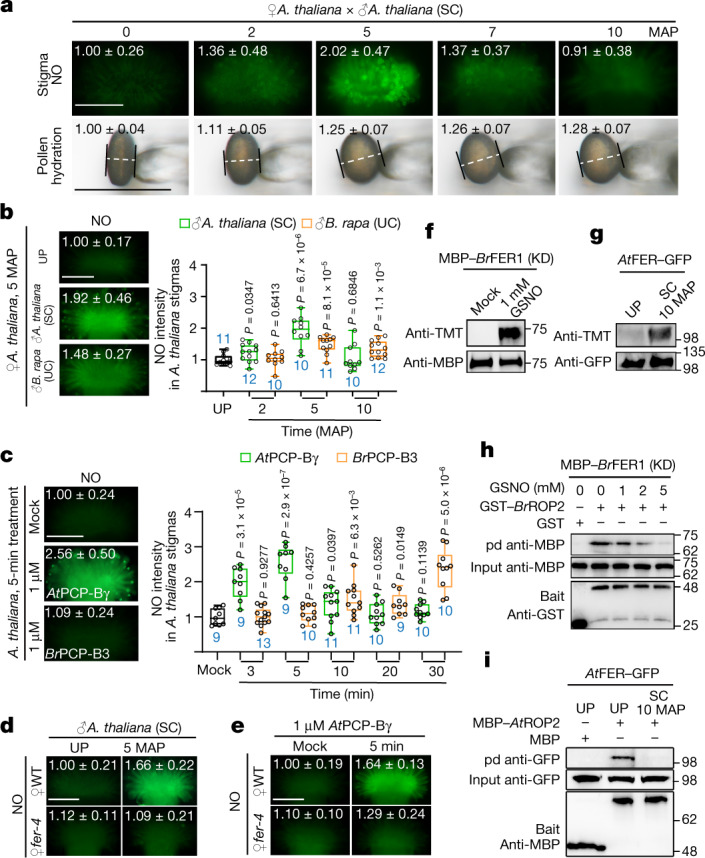
Compatible pollination induces NO to suppress FER-mediated ROS production. **a**, DAF-FM DA staining of pollination-induced NO responses and pollen hydration in *A. thaliana* stigmas. The equatorial diameter of a pollen grain, indicated by white dashed lines, was measured in ImageJ for pollen width. **b**,**c**, Species-preferential elevation of NO in *A. thaliana* stigmas. **d**,**e**, FER-dependent NO elevation in *A. thaliana* stigmas induced by pollen (**d**) and *At*PCP-Bγ (**e**). The values in the **a**–**e** images, shown as average ± s.d., indicate average NO intensity (**a**–**e**) and equatorial diameter of pollen grains (**a**). The same data are also presented in box plots with all data points (Extended Data Fig. 8). **f**,**g**, Nitrosation of FER. *Br*FER1 was nitrosated in vitro by the NO donor GSNO (**f**), and *At*FER–GFP from transformed *A. thaliana* stigmas was nitrosated in vivo by SC pollination (**g**). **h**,**i**, Pull-down assays showing that nitrosation of FER in vitro (**h**) and by pollination (**i**) reduced its interaction with the downstream ROP2 signalling module. The protein samples were derived from the same experiment and the blots were processed in parallel (**f**–**i**). For gel source data, see Supplementary Fig. 1. Scale bars, 200 μm (**a**–**e**) and 50 μm (pollen in **a**). For box plots (**b**,**c**), the centre line indicates the median, the box limits denote the lower and upper quartiles, the dots indicate individual data points, and the whiskers denote the highest and lowest data points. *n* (in blue) indicates the number of stigmas or pollen grains. *P* values were determined by two-tailed Student *t*-tests. Each experiment was repeated at least three times with consistent results. Source data

**Extended Data Fig. 8:**
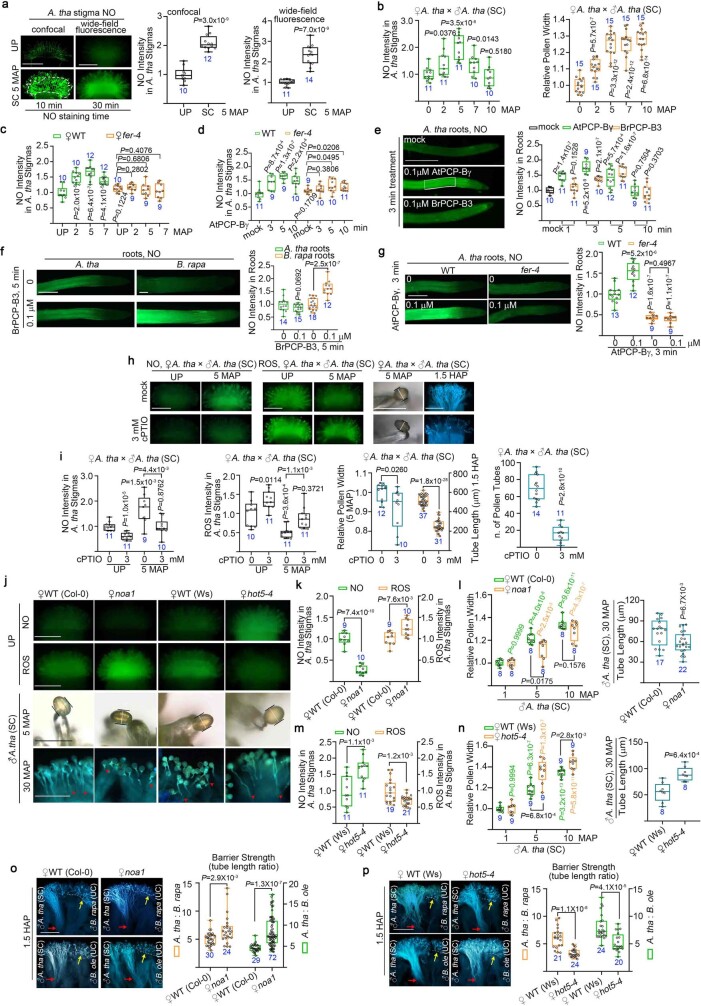
Stigmatic NO is required for ROS reduction in *A. thaliana* stigmas during SC and UC. **a**, **b**, Confocal and wide-field imaging showing the increase of stigmatic NO at 5 MAP with SC pollen (**a**) and quantified data of SC-induced NO and pollen hydration in *A. thaliana* stigmas, both showed NO peaking at ~5 MAP (**b**). See Fig. 4a. **c**, **d**, FER-dependent elevation of NO in *A. thaliana* stigmas induced by pollen and At-PCP-Bγ .See Fig. 4d, e. **e**, AtPCP-Bγ was much faster than GST-BrPCP-B3 in increasing NO of *A. thaliana* roots. **f**, GST-BrPCP-B3 was more effective in increasing NO of *B. rapa* roots than that of *A. thaliana* roots. **g**, Roots of *fer-4* was not responsive to AtPCP-Bγ in inducing NO. **h**, **i**, (Left to right) cPTIO scavenged SC-induced NO, suppressed SC-induced ROS reduction, and inhibited SC pollen hydration and tube growth in *A. thaliana* stigmas. **j**–**n**, Relative to WT, *noa1* stigmas showed lower NO and higher ROS levels, and slower hydration of SC (WT *A. thaliana*) pollen and pollen tube growth (**j**, **k**, **l**). Relative to WT, *hot5-4* stigmas showed faster NO and lower ROS levels, and faster hydration of SC (WT *A. thaliana*) pollen and pollen tube growth (**j**, **m**, **n**). **o**, **p**, Mutations in NOA1, *noa1* (**o**) and GSNOR, *hot5-4* (**p**), enhanced and reduced, respectively, the reproductive barrier to *B. rapa* or *B. oleracea* pollen in *A. thaliana* stigmas at 1.5 HAP. Scale bars, 200 μm (**a**, **h**, **j**, **o**, **p**) and roots (**e**–**g**); 50 μm for pollen (**h**, **j**). Box plots (**a**–**p**): centre line, median; box limits, lower and upper quartiles; dots, individual data points; whiskers, highest and lowest data points. n (in blue), number of stigmas, pollen grains or roots. *P* values, two-tailed *t*-tests. Each experiment was repeated at least thrice with consistent results. Source data

As signalling of ROS and nitric oxide (NO) is intimately engaged^30,31^, we investigated whether NO regulates stigmatic ROS during SC and UC responses. In *A. thaliana* stigmas, NO levels increased rapidly, as early as 2 min after pollination with SC pollen, reaching a maximum by 5 min after pollination, and declined sharply to pre-pollination level, paralleling that of pollen hydration (Fig. 4a, upper panel, and Extended Data Fig. 8a,b). However, *B. rapa* pollen and *Br*PCP-B3 were notably slower than *A. thaliana* pollen and *At*PCP-Bγ, respectively, in stimulating NO in *A. thaliana* stigmas (Fig. 4b,c). Furthermore, unlike wild type, *fer-4* stigma NO was non-responsive to SC pollination and *At*PCP-Bγ treatment (Fig. 4d,e and Extended Data Fig. 8c,d). Species-preferential PCP-B-induced NO increase was also recapitulated in roots (Extended Data Fig. 8e–g). The intraspecific and interspecific pollen and respective PCP-B-induced differential NO increases (Fig. 4b,c) were opposite to their induced changes in ROS (Fig. 3d,e), revealing an inverse functional relationship between NO and ROS in discriminating against interspecific compatible pollen.

**Extended Data Fig. 9:**
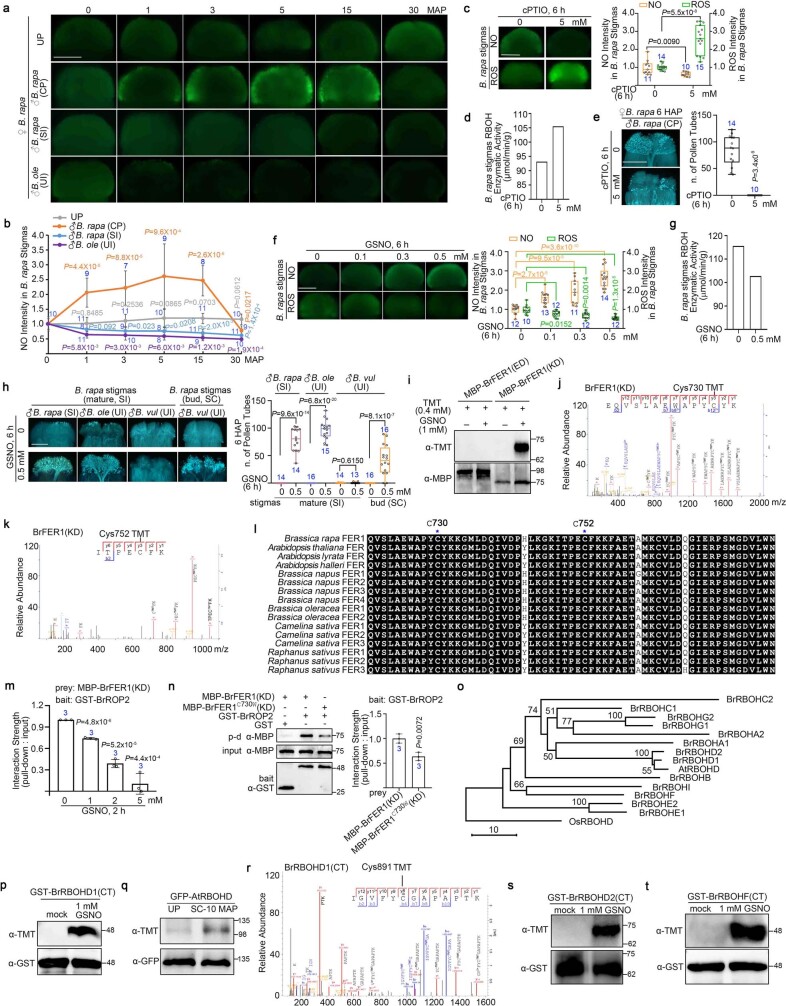
The inverse relationship of NO and ROS and nitrosation of BrFER1 and RBOHs in *B. rapa* stigmas. **a**, **b**, Intraspecific CP pollen (2^nd^ row), but not SI and *B. oleracea* (UI) pollen, induced NO in *B. rapa* stigmas. **c**–**e**, Scavenging NO increased ROS (**c**) and RBOH enzymatic activity of UP *B.rapa* stigmas (**d**), and inhibited the growth of CP pollen tubes in *B. rapa* stigmas (**e**). **f**–**h**, GSNO treatment increased NO (**f**), reduced ROS (**f**) and RBOH enzymatic activity of UP *B. rapa* stigmas (**g**), and promoted the growth of SI or *B. oleracea* pollen in mature or *B. vulgaris* polen in bud-stage *B. rapa* stigmas (**h**). **i**, BrFER1(KD), not BrFER1 (ED), was nitrosated by GSNO. See also Fig. 4f. **j**, **k**, LC-MS spectrum showing the nitrosated cysteine residues in BrFER1 protein. **l**, FER amino acid sequence alignment showing the nitrosated cysteines, Cys730 and Cys752 (*) are evolutionarily conserved. **m**, Pull-down assay shows GSNO treatment induced quantitative inhibition of MBP-BrFER1(KD) with GST-BrROP2 complex. See also Fig. 4h. **n**, The nitrosomimetic mutation, Cys730W, reduced the amount of BrFER1 that was pulled down by GFP-BrROP2. **o**, Phylogenetic analysis of BrRBOHs and AtRBOHD. BrRBOHD1 and BrRBOHD2 are closer to AtRBOHD than other BrRBOHs. **p**–**r**, RBOHD was nitrosated by NO. *In vitro* nitrosation of BrRBOHD1 by GSNO (**p**); CP-induced nitrosation of GFP-AtRBOHD protein from *A. thaliana* stigmas (**q**), and LC–MS spectra showing nitrosation of Cys891 of GST-BrRBOHD1 (CT, C-terminal) (**r**). Nitrosated amino acid residues were labelled with TMT. Proteins were representative of more than three independent preparations. **s**, **t**, GST-BrRBOHD2 (CT) (**s**) and GST-BrRBOHF (CT) (**t**) proteins were nitrosated *in vitro*. The protein samples were derived from the same experiment and the blots were processed in parallel (**i**, **m**, **n**, **p**, **q**, **s**, **t**). For gel source data, see Supplementary Fig. 1. Scale bars, 500 μm. Line chart (**b**): average ± SD. Average relative NO intensities from three biological replicates of stigmas shown in a (two tailed *t*-test, n = 3). Data bars (**d**, **g**): Average activity of ROS producing enzymes from three technical replicates of one stigma sample containing 100 stigmas (two tailed *t*-test, n = 3). Data bar (**m**, **n**): average ± SD. Average relative intensities from three biological replicates of stigmas in (**a**) and blots in Fig 4h and in (**n**) (two tailed *t*-test, n = 3). Box plots (**c**, **e**, **f**, **h**): centre line, median; box limits, lower and upper quartiles; dots, individual data points; whiskers, highest and lowest data points. n (in blue), number of stigmas. *P* values, two-tailed *t*-tests. Each experiment was repeated at least thrice with consistent results unless otherwise specified. Source data

Additional observations further supported the role of NO in regulating stigmatic ROS and pollen growth. Scavenging *A. thaliana* stigma NO by cPTIO^31^ suppressed SC-induced NO increase and ROS reduction and prevented SC pollen growth (Extended Data Fig. 8h,i). Pollination experiments carried out in NO-deficient^32–34^ and NO-overaccumulating^35^ mutants similarly supported the importance of NO involvement (Extended Data Fig. 8j–p). Furthermore, in *B. rapa*, stigmatic NO responded similarly, stimulated by SC but not by SI or UI pollen (Extended Data Fig. 9a,b). Scavenging or increasing NO by cPTIO or *S*-nitrosoglutathione (GSNO), respectively, also induced opposite changes in ROS and pollen growth in *B. rapa* stigmas (Extended Data Fig. 9c–h). These results established firmly that NO is specifically induced in stigmas by compatible pollen for ROS reduction and pollen growth.

## NO nitrosates FER in compatible response

A major bioactivity of NO is *S*-nitrosation of specific cysteines in many proteins^9,36,37^. Given the role of FER and RBOHs examined here (Extended Data Figs. 3 and 4), they could be targets for nitrosation. A tandem mass tag-switch analysis^9,38^ of GSNO-treated MBP–*Br*FER1 showed nitrosation of Cys730, and Cys752 in the KD, which are conserved among FERs from many Brassicaceae species (Fig. 4f and Extended Data Fig. 9i–l). *At*FER–GFP in transformed *A. thaliana* stigmas^8,29^ was also nitrosated after SC pollination (Fig. 4g), suggesting that nitrosation of FER has important roles during the compatible response.

Nitrosation of proteins may affect their stability, biochemical properties and interaction with other proteins^9,39^. GSNO-treated *Br*FER1 (KD) and a Cys730Trp (*Br*FERC730W) conversion, which mimics nitrosation^40^, both showed compromised interaction with its downstream *Br*ROP2 signalling complex (Fig. 4h and Extended Data Fig. 9m,n). We also observed substantial reduction in *At*FER–GFP pulled down by *At*ROP2 in *A. thaliana* stigmas with SC pollen (Fig. 4i). Moreover, stigma-expressed RBOHs were also nitrosated in vitro and by pollination (Extended Data Fig. 9o–t), similar to immunity signalling-induced nitrosation^36^. Together, these results indicate that stigmatic ROS decline is a consequence of compatible pollination-stimulated NO, nitrosating FER to inactivate downstream RAC/ROP-regulated RBOH-dependent ROS production, and the already produced RBOHs to rapidly quell ROS-producing activity in stigmas.

## Breaking the barrier for distant breeding

**Fig. 5:**
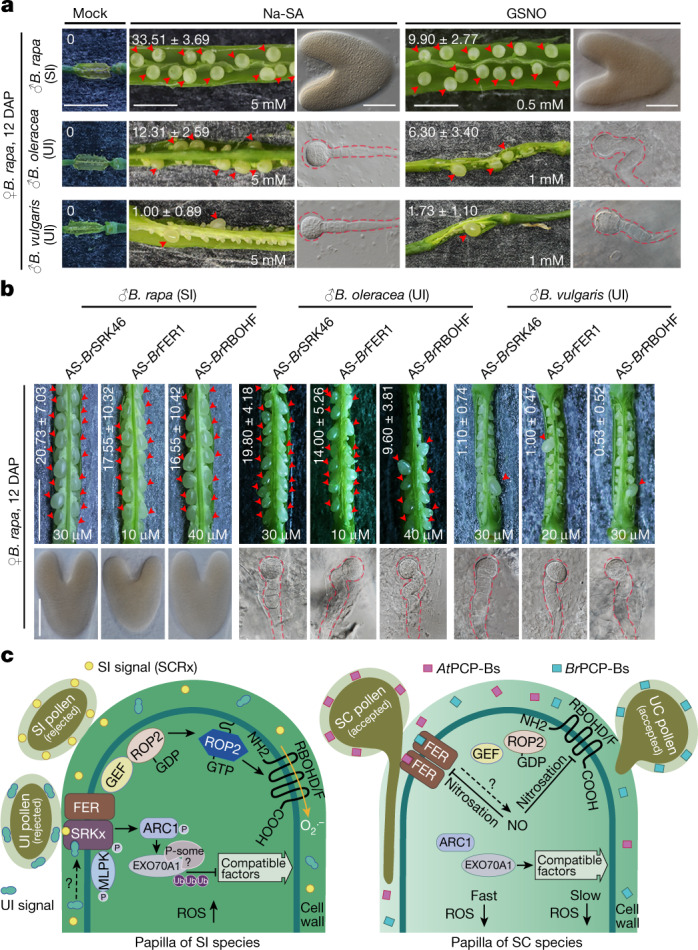
Breaking the stigmatic barrier for distant breeding of Brassicaceae crops. **a**,**b**, Reducing stigmatic ROS by a ROS scavenger, increasing NO by a NO generator (**a**), and disrupting the *Br*SRK–*Br*FER interaction and *Br*FER1-to-*Br*RBOH signalling by AS-ODNs (**b**) alleviated the interspecific and intergeneric reproductive barrier. The arrowheads indicate enlarged ovules. The dashed lines denote the outline of hybrid embryos. The values in the **a**,**b** images, shown as average ± s.d., indicate average number of enlarged ovules in the pod. The same data are also presented in box plots with all data points (Extended Data Fig. 10). Scale bars, 0.5 cm (siliques) and 100 μm (emb ryos). Each experiment was repeated at least three times with consistent results. **c**, Model of FER-regulated ROS as a shared signalling node in SI, UI, SC and UC responses. In stigmas from SI species, SI pollen and UI pollen activate ROS via the SRK–FER–ROP2–RBOHs pathway, functioning along with the ARC1-mediated processes. Stigmas from SC species are compatible to interspecific pollen, but species-preferential interaction between PCP-Bs and FER initiates a faster compatible response via NO-mediated nitrosation of FER and RBOHs to suppress ROS production, promoting intraspecies precedence and protecting species integrity. The dashed lines and ‘?’ indicate ‘to be determined’. P, phosphate; P-some, proteosome; Ub, ubiquitin.

**Extended Data Fig. 10:**
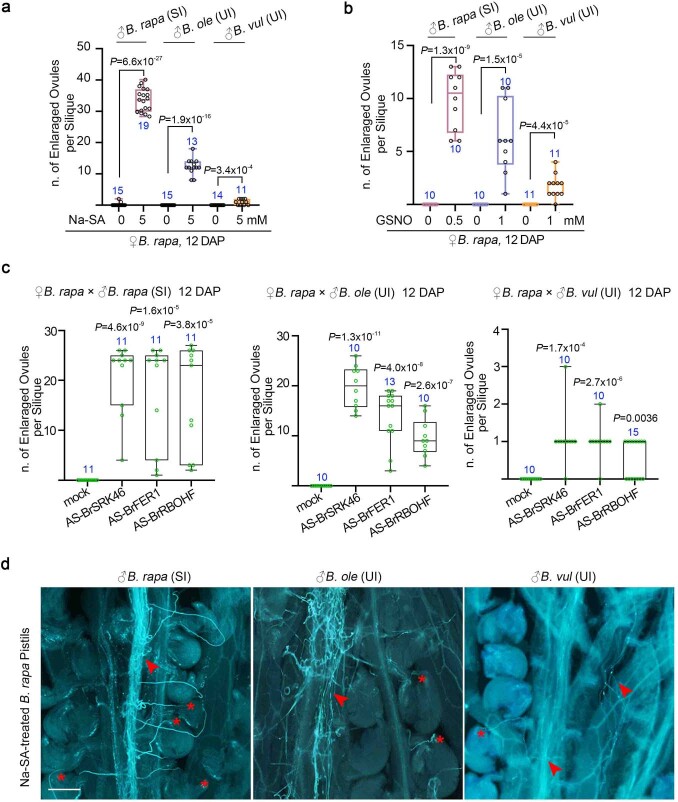
Breaking interspecific barriers in *Brassica* crops. **a**, **b**, Reducing ROS by Na-SA or increasing NO by GSNO increased the number of enlarged ovules in the pod of ♀*B. rapa* × ♂*B. oleracea* or ♀*B. rapa* × ♂*B. vulgaris*. See Fig. 5a. **c**, Suppressing the expression of *BrSRK46*, *BrBrFER1*, or *BrRBOHF* by AS-ODNs increased the number of enlarged ovules in the pod of ♀*B. rapa* × ♂*B. oleracea* or ♀*B. rapa* × ♂*B. vulgaris*. See also Fig. 5b. **d**, *B. oleracea* pollen tubes or *B. vulgaris* pollen tubes in *B. rapa* pistils. The reduced number of enlarged ♀*B. rapa* × ♂*B. vulgaris* ovules is possibly due to fewer *B. vulgaris* pollen tubes had exited the transmitting tissue and targeted the *B. rapa* ovules due to additional barriers based on their evolutionary distance (Fig. 1a). Arrowheads point to bundles of pollen tubes in the transmitting tissue, stars point to ovules penetrated by a pollen tube. Scale bars, 100 μm (**d**). Box plots (**a**–**c**): centre line, median; box limits, lower and upper quartiles; dots, individual data points; whiskers, highest and lowest data points. n (in blue), number of pistils. *P* values, two-tailed *t*-tests. Each experiment was repeated at least thrice with consistent results. Source data

Having established the mechanisms underlying the interspecific barrier at the stigma, we next explored to what extent breaking the barrier might promote distant breeding. We treated *B. rapa* pistils with Na-SA to reduce the levels of ROS and GSNO to increase NO levels, or AS-ODNs to disrupt the *Br*SRK–*Br*FER interaction and *Br*FER-to-*Br*RBOH signalling, then pollinated them with SI and UI pollen. By 12 days after pollination, SI *B. rapa* pollen, *B. oleracea* pollen and *B. vulgaris* pollen all resulted in enlarged ovules with developing embryos in these treated pistils (Fig. 5a,b and Extended Data Fig. 10). Additional barriers beyond the stigma must have precluded robust cross-fertilization and hampered hybrid embryo development (Fig. 5a,b and Extended Data Fig. 10). Combining the strategies used here with embryo rescue, an in vitro culture technique widely utilized in distant breeding^41^, should allow successful development of interspecific hybrid embryos into viable plants.

## Discussion

Upon pollination, pollen and stigma engage in a series of communications to facilitate growth of desirable pollen and discourage invaders and less desirable pollen^1^. Exploring SI, UI, SC and UC together in one study, we demonstrate how the interplay between two receptor kinases, SRK and FER, provides the capacity to perceive different pollen signals to fine tune the stigmatic redox conditions and determine acceptance or rejection of pollen from intraspecies or sympatric interspecies with similar flowering time. As summarized in our working model (Fig. 5c), we demonstrate how incompatible signals from SI and UI pollen activate FER-mediated ROS production for pollen rejection in SI stigmas, functioning in parallel with ARC1-mediated processes^2^. We also demonstrate how the species-preferential interaction of PCP-Bs and FER leads to interspecific barriers in SC stigmas.

The Brassicaceae includes many important vegetable and oil crops. Breeding within a species is far from maximizing hybrid vigour owing to the relatively narrow genetic diversity. Breeding between species enriches germplasm resources but is restricted by interspecific barriers, rendering distant breeding with slim chances of success. Achieving interspecific and intergeneric fertilization here by breaking the stigmatic barrier in *B. rapa*, in particular the production of hybrid embryos with the fungal-resistant and insect-resistant *B. vulgaris*^21,22^ (Fig. 5a,b), represents a remarkable feat and major breakthrough, enabling introgression of desirable traits into crops from distant species.

## Methods

### Plant materials and growth conditions

Stigmas from *B. rapa* var. *pekinensis*, also known as heading Chinese cabbage, and *A. thaliana* were mostly used for pollination responses. For interactions on SI stigmas, *B. rapa* stigmas from a double haploid line of *S*_46_ were pollinated with pollen from *B. rapa S*_46_ or *S*_12_ as SI or CP pollinations, or pollen from *B. oleracea* (*S*_36_), *B. vulgaris* (SC) and *A. thaliana* (SC) as UI pollinations. For interactions on SC stigmas, a variety FPsc with a defective allele of SRK that lacks the coding sequence for the transmembrane domain and SC *A. thaliana* were used. *B. rapa* stigmas from *S*_46,_ *S*_12,_ *S*_9,_ *S*_40_ and *S*_38_ were pollinated with *B. oleracea* (*S*_36_) for haplotype-dependent analysis. Transgenic SI *A. thaliana*^28^, *p*At*FER::*At*FER–GFP*^29^ plants, homozygous *fer-4* (GK-106A06, GABI-Kat)^29^, *srn* (the γ-ray mutant)^42^, *hot5-4* (FLAG_298F11, Versailles Genomic Resource Centre)^35^, *noa1* (CS6511)^9^, *rbohd-3* (Salk_070610C)^10^ and *rbohd-4* (CS9555)^10^ mutants and their corresponding wild-type plants Col-0, C24 or WS have been previously described. Transgenic plants *p*At*FER::GFP–*At*RBOHD* were generated by the *Agrobacterium tumefaciens*-mediated floral dip method^43^. *S*_13_/*fer-4* was generated by crossing *fer-4* mutant into SI Arabidopsis (*S*_13_ of *A. halleri*); / indicates that these two genetic modifications are in the same *Arabidopsis* plant.

Seeds of *B. rapa*, *B. oleracea* and *B. vulgaris* were germinated in potted soil (Pindstrup substrate, Denmark). Vernalization was performed in a growth chamber with 10 °C–5 °C, 14 h–10 h light–dark cycles, and light intensity of 100 mmol m^−^^2^ s^−1^. After 1 month of cold treatment for 1-week-old *B. rapa*, and 3 months for 7–8 leaf stage *B. oleracea* and *B. vulgaris* plants, these plants were planted in soil under greenhouse conditions with 25 °C–15 °C, 16 h–8 h light–dark cycles, and light intensity of 300 mmol m^−^^2^ s^−1^. Seeds of *A. thaliana* and *Nicotiana* *benthamiana* were germinated and grew in potted soil in a greenhouse at 22 °C, 16–8 h light–dark cycle with a relative humidity of 60%.

### Statistical analysis

Data involving ROS, NO, pollen hydration and pollen tube length were presented as box plots generated in GraphPad Prism v8.0.1. Unless otherwise indicated, the centre line of box plots denotes the median, the box limits denote the lower and upper quartiles, and the whiskers denote the lowest and highest data points. Protein plots and quantitative PCR with reverse transcription (qRT–PCR) related data were presented as data bars (average ± s.d., *n* = 3). Data involving changes of stigma NO over time after pollination were presented as a line chart (average ± s.d.; *n* indicates the number of stigmas). All statistics data were labelled with the exact *P* value, and dots in data bars and box plots denote individual data points. Each experiment was repeated at least three times with consistent results.

### Stigma treatment and pollen growth observation

Compatibility was demonstrated by the number of pollen tubes that had penetrated the stigma papilla cells. Stigma feeding assays followed that of refs. ^10,44^. *B. rapa* flowers that have just opened but before anther dehiscence, or bud-stage flowers were emasculated and cut at 3 mm away from the stigmatic surface. Excised stigmas were inserted into basic PGM (5 mM CaCl_2_, 5 mM KCl, 0.01% H_3_BO_3_, 1 mM MgSO_4_•7H_2_O, 10% sucrose and 0.8% agarose, pH 7.5) or the treatment medium and kept in a chamber with constant temperature (22.5 °C) and humidity (45%) for the indicated period of time (S-ODNs or AS-ODNs for 1 h; Na-SA, GSNO and cPTIO for 6 h). Treated stigmas were transferred to basic PGM medium and manually pollinated with similar amount of SI, CP or various UI pollen and maintained in the same condition for 6 h or as indicated. Stigmas were then fixed in Canoy’s fixative (ethanol to acetic acid 3:1), softened in 10 M NaOH, and stained in 0.1% aniline blue. Pollen tubes were visualized by epifluorescence (Ex375-328, DM415 and BA351p) on a Nikon Eclipse Ni. Images were captured by a DS-Ri2 digital camera.

For in planta treatment, just open flowers, or bud-stage flowers, on inflorescences of *B. rapa* plants were treated twice at a 30-min interval, with S-ODN and AS-ODN, Na-SA, GSNO or the corresponding mock solution, supplemented with 0.0125% Tween, then pollinated with a similar amount of SI, CP, *B. oleracea* or *B. vulgaris* pollen. The number of enlarged ovules was counted at 12 days after pollination. Embryo clearing and observation followed that of ref. ^45^ with modifications. Enlarged ovules were cleared in Hoyer’s medium (7.5 g gum arabic, 100 g chloral hydrate, 5 ml glycerol and 60 ml H_2_O) for 5 days, then observed under differential interference contrast on a Nikon Eclipse Ni microscope equipped with a DS-Ri2 digital camera.

The effect of chemicals or the mutation of stigma-expressed genes on the growth of intraspecific or interspecific pollen on SC *A. thaliana* stigmas was demonstrated by the rate of pollen hydration or pollen tube length. SC *A. thaliana* flowers were emasculated at stage 12 (ref. ^46^), cultured in PGM for 14 h, then pollinated with *A. thaliana* pollen or *B. rapa* pollen. For pollen hydration, images were taken at each time point after pollination under differential interference contrast on a Nikon Eclipse Ni microscope equipped with a DS-Ri2 digital camera. The equatorial diameters of pollen grains at various time points were measured in ImageJ v1.53c. For pollen tube length, *A. thaliana* stigmas with *A. thaliana* pollen, *B. rapa* pollen or *B. oleracea* pollen were processed for aniline blue staining at 1 or 1.5 HAP. In dual-pollination assays, *A. thaliana* WT and *fer*-mutant pistils (or other mutant pistils) were simultaneously pollinated with *A. thaliana* pollen on half and either *B. rapa* or *B. oleracea* pollen on the other half of the same stigma, with a clear boundary in between. Pollen growth from each half of the stigma was clearly confined to the corresponding half of the pistils and readily distinguishable.

### ODN design and treatment

ODN design and treatment of stigmas followed that of ref. ^10^. S-ODNs and AS-ODNs were used to target the following genes (accession numbers shown in Supplementary Table 1): *Br*SRK46, *Br*FER1 and *Br*RBOHF. S-ODNs or AS-ODNs were designed based on Sfold (https://sfold.wadsworth.org/cgi-bin/soligo.pl). The BLAST program (https://blast.ncbi.nlm.nih.gov/Blast.cgi) was used to assess potential off-target effect. The ODNs were synthesized in the Beijing Genomics Institution (BGI). Three bases at both 5′ and 3′ end of S-ODN and AS-ODN were phosphorothioate-modified to maintain stability. The sequences of S-ODNs and AS-ODNs were listed in Supplementary Table 2. Stigmas were excised at the style 1 mm away from the top, inserted in PGM containing the S-ODN or AS-ODN and treated for 1 h. Stigmas were subjected to aniline blue assay at 6 HAP to observe pollen growth.

### Staining of ROS and NO

For stigmatic ROS staining, *B. rapa* stigmas or *A. thaliana* stigmas, unpollinated or at 10 min after pollination with SI, CP or UI pollen, were pretreated in MES buffer (10 mM MES, 5 μM KCl and 50 μM CaCl_2_, pH 6.15) for 30 min, stained with 50 μM H_2_DCF-DA (2′,7′-dichlorofluorescein diacetate; Sigma-Aldrich) for 30 min, respectively, then washed at least three times in buffer before observation. For stigmatic NO staining, *B. rapa* stigmas or *A. thaliana* stigmas before or after pollination were soaked in Tris buffer (10 mM Tris-HCl and 10 mM KCl, pH 7.5) for 30 min, stained with 20 μM DAF-FM DA (3-amino,4-aminomethyl-2′,7′-difluorescein diacetate; Thermo Scientific) for 1 h, then washed at least three times in buffer before observation.

For treatments, stigmas were first soaked in MES buffer for 30 min, then treated with S-ODNs or AS-ODNs, chemicals, peptides or pollen extracts at indicated concentration and duration in MES buffer supplemented with 0.0125% Tween 20. After washing three times in MES buffer, treated stigmas were stained for ROS or NO as above described.

For root ROS or NO staining, 3-day-old *A. thaliana* seedlings or 2-day-old *B. rapa* seedlings were soaked in the corresponding buffer for 30 min, treated with 0.1 μM of *At*PCP-Bγ or *Br*PCP-B3 for the indicated period of time, then stained with 50 μM H_2_DCF-DA or 20 μM DAF-FM DA for 1 h.

Imaging was carried out under eGFP epifluorescence (Ex470-440, DM4951p and BA525/550), using a Nikon Eclipse Ni and equipped with a DS-Ri2 digital camera. The exposure time for all comparative samples were exactly the same within one experiment. Average ROS or NO signals outlined in the dotted area were measured in Image J v1.53c; ROS or NO in control stigmas were set at 1 for comparative analyses.

For comparison, stigmas were directly stained with the same concentrations of H_2_DCF-DA or DAF-FM DA for 10 min without buffer pretreatment, and imaged under a confocal microscope (Zeiss LSM880). Changes of ROS and NO were consistent with those imaged under wide-field fluorescence microscope. Wide-field observation, due to its considerably high expedition to sample entire stigma specimens, was used for the massive amount of data gathering required for this study.

For ROS staining of infiltrated tobacco leaves, agrobacterium cells containing *Br*FER1–MYC, GFP–*Br*RBOHD2 and *Br*SRK46–HA were mixed and infiltrated into tobacco leaves. Two days after infiltration, 10 μM GST–*Br*SCR12 or GST–*Br*SCR46 were injected into the same leaf and treated for 10 min. Nitro blue tetrazolium (NBT) staining^47^ was used to detect ROS of the infiltrated leaves. The leaves were vacuum infiltrated with 5 mg ml^−1^ NBT (in 10 mM sodium citrate, pH 7.0) for 40 min at room temperature. Leaves were then decolourized three times by boiling in decolourized solution (95% ethanol to glycerine 3:1) for 10 min. The images were captured by a digital camera and measured for ROS intensity in Image J v1.53c. For ROS staining of protoplasts, protoplasts were isolated from the above infiltrated tobacco leaves co-expressing *Br*FER1–MYC, GFP–*Br*RBOHD2 and *Br*SRK46–HA, following that of ref. ^48^. Protoplasts were then treated with buffer, *Br*SCR12 and *Br*SCR46, respectively, for 15 min, before staining with 10 μM H_2_DCF-DA for 10 min. Protoplasts from tobacco leaves infiltrated with buffer were stained for ROS as a control.

### Enzymatic activity of RBOHs

Stigmas before or after pollination or treatment were washed three times with the MES buffer and then freezed in liquid N_2_. Enzymatic activity of RBOHs was measured following the manufacturer’s instructions (Nanjing JC bio). In brief, approximately 0.05 g stigma tissue (approximately 100 stigmas) was ground in liquid N_2_, extracted in buffer (0.2 M NaH_2_PO_4_ and 0.2 M Na_2_HPO_4_, pH 7.2) and centrifuged at 4,000*g* for 20 min at 4 °C. The resulting supernatant was used to measure RBOH activity spectrophotometrically at 340 nm using FAD and NADPH as substrates. The result was shown as the average of three technical replicates of one sample.

### RNA isolation and qRT–PCR

Total RNA was extracted via the SteadyPure Universal RNA Extraction Kit (AG21017, Accurate Biotechnology) and reverse transcribed with HiScript III 1st Strand cDNA Synthesis Kit (Vazyme). qRT–PCR was performed on a QuantStudio 3 system (Applied Biosystems) with ChamQ SYBR qPCR Master Mix (Vazyme), using Br*ACTIN2* as internal controls. A list of gene-specific primers used for qRT–PCR is included in Supplementary Table 3.

### Molecular cloning

For the *Br*SRK46–HA or *Br*RBOHD2–GFP construct, sequences encoding the full length of *Br*SRK46 or *Br*RBOHD2 were amplified from *B. rapa* stigma cDNA using 2× Phanta Max Master Mix (Vazyme). The fragment was cloned into the modified pCAMBIA1300 vector with a HA or GFP tag using pEASY-Basic Seamless Cloning and Assembly Kit (TransGen Biotech). For the GST fusion protein constructs, sequences encoding *Br*PCP-B3 (residues 1–76), *Br*SCR46 (residues 1–78), *Br*SCR12 (residues 1–76), full length of *Br*ROP2, C-terminal cytoplasmic region of *Br*RBOHD1 (CT; residues 758–923), *Br*RBOHD2 (CT; residues 614–904) and *Br*RBOHF (CT; residues 767–949) were amplified and cloned into a pGEX-4T-1 vector. For the MBP fusion protein constructs, sequences encoding ED of *Br*FER1 (residues 14–420), KD of *Br*FER1 (residues 486–783), mature *At*PCP-Bγ (residues 23–76) and *At*ROP2 were amplified and cloned into a pMAL-p2X vector. For the FLAG fusion protein construct, the ED of *At*FER (residues 29–446) and the KD of *Br*SRK46 (residues 448–860) were amplified and cloned into the pFLAG-CTS vector. For the MYC fusion protein construct, *Br*FER1 (residues 1–788) was amplified and cloned into pCXSNF. For the bimolecular fluorescent complementation constructs, the KD of *Br*SRK46 (residues 448–860) and *Br*FER1 (residues 486–783) were amplified and cloned into pDONR207 by the Gateway BP reaction via Gateway BP Clonase Enzyme Mix (Invitrogen Life Technologies) and cloned into the pEARLY GATE 201 vector or pEARLY GATE 202 by the LR reaction via Gateway LR Clonase Enzyme Mix (Invitrogen Life Technologies). For the yeast two-hybrid constructs, the KD of *Br*SRK46 (residues 448–860) and the KD of *Br*FER1 (residues 486–783) were amplified and cloned into the pGADT7 or pGBKT7 vectors. For MBP–*Br*FER1^C730W^ (KD), the mutant fragment was amplified from the MBP–*Br*FER1 (KD) vector with appropriate PCR primers using the Fast Mutagenesis System (TransGen Biotech) according to the manufacturer’s instructions. A list of gene-specific primers used for the above constructs is included in Supplementary Table 4.

### Protein expression and purification

The constructs of GST, MBP and FLAG fusion protein were transformed into *Escherichia coli* BL21 (DE3) for protein expression. After induction by 0.5 mM IPTG at 37 °C for 4–6 h, the cells were spun down and resuspended in 5 ml PBS (140 mM NaCl, 2 mM KCl, 2 mM KH_2_PO_4_ and 10 mM Na_2_HPO_4_.7H_2_O). Sonicated (SCIENTZ) protein was purified by magnetic GSH beads (BEAVER), amylose magnetic beads (PuriMag Pro) or anti-FLAG magnetic beads (BeyoMag), respectively. The eluted protein was separated by SDS–PAGE and detected by the corresponding antibody after western blot.

For peptide preparation, MBP–*At*PCP-Bγ and GST–*Br*PCP-B3 were expressed in *E. coli* BL21 cells and purified by corresponding magnetic beads. The *At*PCP-Bγ (residues 23–76) peptides were also synthesized by Scilight Biotechnology with more than 95% of purity. The peptides were diluted to 1 mM in sterile ddH_2_O as stock solution.

For proteins expressed in tobacco leaves, *Agrobacterium tumefaciens* GV3101 (Weidi) cells containing corresponding constructs were infiltrated into *N. benthamiana* leaves following standard procedure^48^. In brief, *Agrobacterium* cells were spun down at 5,000*g* for 10 min at 4 °C and resuspended to an optical density at 600 nm of 0.6 in the infiltration buffer (10 mM MES, 10 mM MgCl_2_ and 0.5% glucose, pH 6.5), with 100 μM acetosyringone added before infiltration. Leaves from 5–6-week-old tobacco plants were infiltrated using a 1-ml syringe. Two days after infiltration, leaves were homogenized in liquid N_2_, mixed in 1 ml plant protein extraction buffer (75 mM KAc, 300 mM NaCl, 100 mM arginine, 10 mM EDTA, 0.25% Triton X-100, pH 7.4, 1 mM PMSF and 1 mM cocktail protease inhibitor) in a 1.5-ml centrifuge tube and stayed on ice for 20 min. After centrifuge at 13,800*g* for 10 min at 4 °C, the supernatant was transferred into a new tube for pull-down or co-IP assays.

### Protein interaction assays

For the yeast two-hybrid assay of *Br*FER1 (KD) and *Br*SRK46 (KD), vector construction, yeast transformation, growth on drop out medium and X-Gal staining followed standard procedure^29^.

For the bimolecular fluorescent complimentary assay, the pEARLY GATE 201 containing *Br*FER1 (KD)–nYFP and the pEARLY GATE 202 containing *Br*SRK46 (KD)–cYFP were transformed into *Agrobacterium tumefaciens* strain GV3101. Equal volumes of two cultures were mixed and infiltrated into *N. benthamiana* leaves as described above. Two days after infiltration, imaging was carried out under eGFP epifluorescence (Ex470-440, DM4951p and BA525/550), using a Nikon Eclipse Ni and equipped with a DS-Ri2 digital camera.

For the pull-down assay of *Br*SRK46–HA by MBP–*Br*FER1 (KD), *Br*SRK46–HA protein was extracted from infiltrated tobacco leaves, mixed with amylose magnetic beads (PuriMag Pro)-bound MBP–*Br*FER1 (KD) bait protein for 6 h at 4 °C. The beads were washed three times in pull-down buffer (50 mM Tris-HCl, pH 7.0, 100 mM NaCl and 0.1% Triton X-100 (v/v)) and boiled in SDS–PAGE loading buffer for 5 min. The proteins were then processed for SDS–PAGE, western blot and immunodetection by anti-MBP antibody (1:15,000; Abmart) or anti-HA antibody (1:5,000; Abmart), and with anti-mouse horseradish peroxidase (HRP)-conjugated secondary antibody (1:15,000; Abmart) followed by the HRP detection kit (Vazyme) analysis with a chemiluminescence imaging system (TIAN NENG). For the effect of peptides, GST–*Br*SCR46 or GST–*Br*SCR12, or protein extracts from SI (*S*_46_), CP (*S*_12_) pollen or interspecific (*B. oleracea*) pollen on the interaction of *Br*SRK46–*Br*FER1 (KD), 0.5 μM peptides or 0.65 mg ml^−1^ protein extracts were incubated with *Br*SRK46–HA protein for 1 h before the pull-down assay.

For the co-IP assay of *Br*FER1–MYC by *Br*SRK46–HA, GV3101 *Agrobacterium* cells containing *35S::*Br*SRK46–HA* or *35S::*Br*FER1–MYC* were mixed and infiltrated together into *N. benthamiana* leaves. Two days after infiltration, 10 μM GST–*Br*SCR12 or GST–*Br*SCR46 were infiltrated into the same leaf and treated for 10 min. The leaves were then used for protein extraction. Aliquots of 1 ml supernatant were incubated with 100 μl anti-HA magnetic beads (PuriMag Pro) at 4 °C overnight with rotation. After washing three times, the beads were boiled and the proteins were processed for SDS–PAGE, western bolt and immunodetection by anti-MYC antibody (1:5,000; Abmart) and anti-HA antibody (1:5,000; Abmart) and anti-mouse HRP-conjugated secondary antibody (1:10,000; Abmart).

For the pull-down assay of MBP–*Br*FER1 (KD) by GST–*Br*ROP2, MBP–*Br*FER1 (KD) protein was treated with 1, 2 and 5 mM GSNO for 3 h at room temperature, then mixed with GSH bead (BEAVER)-bound, GST–*Br*ROP2 bait protein for 6 h at 4 °C. The beads were boiled and processed for SDS–PAGE, western bolt and immunodetection by anti-MBP antibody (1:15,000; Abmart) or anti-GST antibody (1:5,000; Abmart), and with anti-mouse HRP-conjugated secondary antibody (1:15,000; Abmart). MBP–*Br*FER1^C730W^ (KD), without GSNO treatment, was processed similarly for the pull-down assay with GST–*Br*ROP2.

For the pull-down of *At*FER–GFP by MBP–*At*ROP2, MBP or MBP–*At*ROP2 bait proteins were bound with amylose magnetic beads (PuriMag Pro) for 6 h and washed three times with pull-down buffer. *A. thaliana* stigmas from *p*At*FER::*At*FER–GFP* transgenic plants, unpollinated or 10 min after pollination, were used for total protein extraction. *At*FER–GFP protein was incubated with corresponding bait protein for 8 h at 4 °C. After washing three times, the beads were boiled and processed for SDS–PAGE, western bolt and immunodetection by anti-GFP antibody (1:10,000; Abmart) or anti-MBP antibody (1:15,000; Abmart), and with anti-mouse HRP-conjugated secondary antibody (1:10,000; Abmart).

For the pull-down assay to examine peptide competition, anti-FLAG magnetic bead (BeyoMag)-bound *At*FER (ED)–FLAG bait protein was incubated with GST–*Br*PCP-B3 with rotation at 4 °C for 2 h, then increasing concentrations of *At*PCP-Bγ peptides were added for competition at 4 °C for 3 h. The competition by GST–*Br*PCP-B3 peptides with MBP–*At*PCP-Bγ in interaction with *At*FER (ED)–FLAG bait protein was performed similarly. The beads were boiled and processed for SDS–PAGE, western bolt and immunodetection by anti-FLAG antibody (1:5,000; Abmart), anti-GST antibody (1:5,000; Abmart) or anti-MBP antibody (1:15,000; Abmart), and anti-mouse HRP-conjugated secondary antibody (1:5,000; Abmart).

### Protein nitrosation assay

In vitro *S*-nitrosation assay followed that of ref. ^35^ with modifications, using Pierce *S*-Nitrosylation Western Blot Kit (90105, Thermo Scientific). Purified proteins of GST–*Br*RBOHD1 (CT), GST–*Br*RBOHD2 (CT), GST–*Br*RBOHF (CT) and MBP–*Br*FER1 (KD) were desalted by acetone precipitation and resuspended in HENS buffer (100 mM HEPES, pH 7.0, 1 mM EDTA, 0.1 mM neocuproine and 2.5% SDS). Approximately 150 μg proteins per sample were incubated with 1 mM GSNO in a reaction volume of 100 μl HENS buffer for 2 h at room temperature in the dark. The sample was precipitated with cold acetone and resuspended in 100 μl HENS buffer. After incubation at 50 °C for 1 h with 200 mM *N*-ethylmaleimide (Solarbio) and at room temperature for 30 min, the sample was precipitated with cold acetone and resuspended in HENS buffer. The sample was treated with 60 mM sodium ascorbate and 0.4 mM iodoTMTzero label reagent for 2 h. All the above steps were carried out in the dark. The proteins were finally precipitated by cold acetone and resuspended in 2 M urea buffer. Aliquots of each protein were separated by SDS–PAGE and then analysed by immunoblotting with anti-TMT antibody (1:5,000; Thermo Scientific) and goat anti-mouse IgG (H+L)–HRP (1:10,000; Thermo Scientific) were used for detection of nitrosated protein. In parallel, the proteins were detected for loading with anti-GST antibody (1:5,000; Abmart) or anti-MBP antibody (1:15,000; Abmart), and goat anti-mouse IgG–HRP (1:10,000; Abmart).

For the analysis of *S*-nitrosation in stigmas before and after pollination, 200 stigmas (approximately 0.1 g) from *A. thaliana* plants expressing *p*At*FER::*At*FER–GFP* or *p*At*FER::GFP–*At*RBOHD*, unpollinated or at 10 min after pollination with intraspecific compatible pollen (WT *Arabidopsis* pollen), were homogenized in liquid N_2_ and resuspended in 0.7 ml of the plant protein extraction buffer. Protein extracts, 1.5 mg in 150 μl, were precipitated with cold acetone and resuspended in HENS buffer for the detection of nitrosated proteins similar to in vitro nitrosation detection.

### Mass spectrometry analysis of nitrosated residue

Mass spectrometric identification of *S*-nitrosated cysteine residues was carried out by Shanghai Bioprofile Biotechnology (China). The protein pellets were resolved with HENS buffer and *N*-ethylmaleimide (Sigma) was used to block the free cysteine. The *S*-nitrosation sites of protein were reduced by the sodium ascorbate (Sigma) specifically and then labelled with iodoTMT zero reagent (Thermo Scientific). The processed proteins were digested with trypsin in 50 mM NH_4_HCO_3_ overnight at 37 °C. The peptides were then desalted with C18 cartridge (Thermo Scientific). The iodoTMT-labelled peptides were enriched by anti-TMT resin as instructed by the manufacturer (Thermo Scientific). Then, the enriched peptides were loaded into liquid chromatography–mass spectrometry for analysis. The Q Exactive HF-X mass spectrometer coupled to Easy nLC1200 (Thermo Scientific) were performed on a 2-h time gradient for peptide mass spectrometry detection. The mass spectrometry raw data were imported into MaxQuant software v1.6.0.16 for data interpretation and protein identification against the *B. rapa* genome database^49^ (http://brassicadb.cn). The search results were filtered and exported with a less than 1% false discovery rate at the site level, peptide-spectrum-matched level and protein level. MaxQuant analysis was filtered only for those nitrosated sites (iodoTMT labelled) that were confidently localized (class I, localization probability of more than 0.75) and the score of the modified peptide was more than 40.

### Bioinformatic analysis

All sequences analysed were retrieved from the *B. rapa* genome database^49^ (http://brassicadb.cn), NCBI GenBank^50^ (https://www.ncbi.nlm.nih.gov/genbank) or EnsemblPlants^51^ (http://plants.ensembl.org/index.html) or Phytozome^52^ (https://phytozome-next.jgi.doe.gov). *C. danica* was selected as the outgroup to infer the species relationships among *B. rapa*, *B. oleracea*, *B. vulgaris*, *A. thaliana*, *Raphanus sativus*, *Arabidopsis lyrata* and *A. halleri*. The maximum likelihood tree based on the alignment of nuclear ribosomal internal transcribed spacers from the above-mentioned species was inferred under a Tamura–Nei nucleotide substitution model with 1,000 bootstraps. For the phylogenetic tree of RBOHs, PCPs and SRKs, corresponding sequences were aligned using the MUSCLE algorithm implemented in MEGA X^53^, and constructed using the neighbour-joining method in MEGA X with 1,000 bootstraps. The amino acid sequence of PCP-Bs and FER alignment was performed by the online software Clustal Omega^54^ (https://www.ebi.ac.uk/Tools/msa/clustalo) with default parameters then import into ESPript 3.0 (ref. ^55^) (https://espript.ibcp.fr/ESPript/cgi-bin/ESPript.cgi) to generate pictures.

### Figure preparation

Average ROS and NO signals, pollen grain width and pollen tube length were measured by Image J v1.53c (https://imagej.net). Histograms were prepared by GraphPad Prism v8.0.1 (https://www.graphpad-prism.cn). The main figures were assembled in Adobe Illustrator; all other figures were assembled in Adobe Photoshop. Some cartoon components were from www.figdraw.com for model drawing.

### Reporting summary

Further information on research design is available in the Nature Portfolio Reporting Summary linked to this article.

## Online content

Any methods, additional references, Nature Portfolio reporting summaries, source data, extended data, supplementary information, acknowledgements, peer review information; details of author contributions and competing interests; and statements of data and code availability are available at 10.1038/s41586-022-05640-x.

## Supplementary information

Supplementary Information This file contains Supplementary Fig. 1 and Supplementary Tables 1–4.

Reporting Summary

## Data Availability

The source data for Figs. 1–5 and Extended Data Figs. 1–10 are provided with the paper. Raw, uncropped gels and gene accession numbers used in this paper are shown in the Supplementary Information. Source data are provided with this paper.

## Source data

Source Data Fig. 3
Source Data Fig. 4
Source Data Extended Data Fig. 1
Source Data Extended Data Fig. 2
Source Data Extended Data Fig. 3
Source Data Extended Data Fig. 4
Source Data Extended Data Fig. 5
Source Data Extended Data Fig. 6
Source Data Extended Data Fig. 7
Source Data Extended Data Fig. 8
Source Data Extended Data Fig. 9
Source Data Extended Data Fig. 10
