# Author Correction: Stigma receptors control intraspecies and interspecies barriers in Brassicaceae

**DOI:** 10.1038/s41586-023-05816-z

**Published:** 2023-03-02

**Authors:** Jiabao Huang, Lin Yang, Liu Yang, Xiaoyu Wu, Xiaoshuang Cui, Lili Zhang, Jiyun Hui, Yumei Zhao, Hongmin Yang, Shangjia Liu, Quanling Xu, Maoxuan Pang, Xinping Guo, Yunyun Cao, Yu Chen, Xinru Ren, Jinzhi Lv, Jianqiang Yu, Junyi Ding, Gang Xu, Nian Wang, Xiaochun Wei, Qinghui Lin, Yuxiang Yuan, Xiaowei Zhang, Chaozhi Ma, Cheng Dai, Pengwei Wang, Yongchao Wang, Fei Cheng, Weiqing Zeng, Ravishankar Palanivelu, Hen-Ming Wu, Xiansheng Zhang, Alice Y. Cheung, Qiaohong Duan

**Affiliations:** 1 State Key Laboratory of Crop Biology, Shandong Agricultural University, Tai’an, China; 2 College of Horticulture Science and Engineering, Shandong Agricultural University, Tai’an, China; 3 Institute of Horticulture, Henan Academy of Agricultural Sciences, Zhengzhou, China; 4 Computer Network Information Centre, Chinese Academy of Sciences, Beijing, China; 5 National Key Laboratory of Crop Genetic Improvement, Huazhong Agricultural University, Wuhan, China; 6 Shandong Yiyi Agricultural Science and Technology Co., Ltd, Tai’an, China; 7 College of Horticulture, Qingdao Agricultural University, Qingdao, China; 8 International Flavors & Fragrances, Wilmington, DE USA; 9 School of Plant Sciences, University of Arizona, Tucson, AZ USA; 10 Department of Biochemistry and Molecular Biology, Molecular Cell Biology and Plant Biology Programs, University of Massachusetts, Amherst, MA USA

**Keywords:** Plant signalling, Fertilization

Correction to: *Nature* 10.1038/s41586-022-05640-x Published online 25 January 2023

In the version of this article initially published, the affiliation listed for Weiqing Zeng was incorrect (Trait Discovery, Corteva Agriscience, Johnston, IA, USA); it should have read International Flavors & Fragrances, Wilmington, DE, USA. The affiliation has been corrected in the HTML and PDF versions of the article.

