# Supplementary material for: Stigma receptors control intraspecies and interspecies barriers in Brassicaceae
Source: Nature. 2023 Jan 25;614(7947):303–8. doi: 10.1038/s41586-022-05640-x (PMC9908550; doi:10.1038/s41586-022-05640-x)
Supplement: Supplementary file 1 — This file contains Supplementary Fig. 1 and Supplementary Tables 1–4. [file 41586_2022_5640_MOESM1_ESM.pdf]

nature portfolio

https://doi.org/10.1038/s41586-022-05640-x

Supplementary information

Stigma receptors control intraspecies and interspecies barriers in Brassicaceae

In the format provided by the authors and unedited

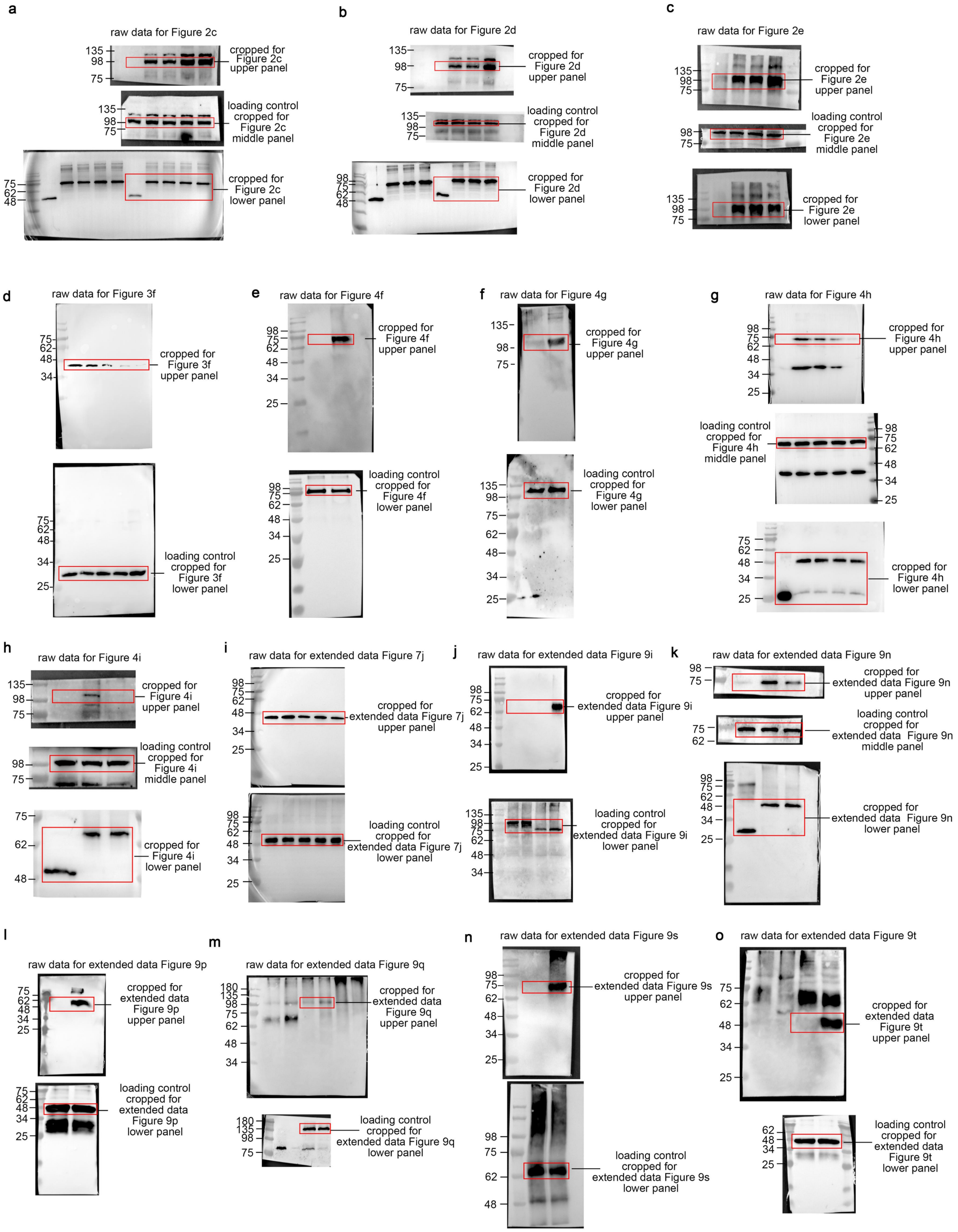
a
raw data for Figure 2c
cropped for Figure 2c upper panel
loading control cropped for Figure 2c middle panel
cropped for Figure 2c lower panel
b
raw data for Figure 2d
cropped for Figure 2d upper panel
loading control cropped for Figure 2d middle panel
cropped for Figure 2d lower panel
c
raw data for Figure 2e
cropped for Figure 2e upper panel
loading control cropped for Figure 2e middle panel
cropped for Figure 2e lower panel
d
raw data for Figure 3f
cropped for Figure 3f upper panel
loading control cropped for Figure 3f lower panel
e
raw data for Figure 4f
cropped for Figure 4f upper panel
loading control cropped for Figure 4f lower panel
f
raw data for Figure 4g
cropped for Figure 4g upper panel
loading control cropped for Figure 4g lower panel
g
raw data for Figure 4h
cropped for Figure 4h upper panel
loading control cropped for Figure 4h middle panel
cropped for Figure 4h lower panel
h
raw data for Figure 4i
cropped for Figure 4i upper panel
loading control cropped for Figure 4i middle panel
cropped for Figure 4i lower panel
i
raw data for extended data Figure 7j
cropped for extended data Figure 7j upper panel
loading control cropped for extended data Figure 7j lower panel
j
raw data for extended data Figure 9i
cropped for extended data Figure 9i upper panel
loading control cropped for extended data Figure 9i lower panel
k
raw data for extended data Figure 9n
cropped for extended data Figure 9n upper panel
loading control cropped for extended data Figure 9n middle panel
cropped for extended data Figure 9n lower panel
l
raw data for extended data Figure 9p
cropped for extended data Figure 9p upper panel
loading control cropped for extended data Figure 9p lower panel
m
raw data for extended data Figure 9q
cropped for extended data Figure 9q upper panel
loading control cropped for extended data Figure 9q lower panel
n
raw data for extended data Figure 9s
cropped for extended data Figure 9s upper panel
loading control cropped for extended data Figure 9s lower panel
o
raw data for extended data Figure 9t
cropped for extended data Figure 9t upper panel
loading control cropped for extended data Figure 9t lower panel

Supplementary Figure 1. Raw, uncropped data for gels for each data were presented in the same order as in figures. Fig. 2e (a), Fig. 2f (b), Fig. 2g (c), Fig. 3f (d), Fig. 4h (e), Fig. 4i (f), Fig. 4j (g), Fig. 4k (h), Fig. 7j (i), Fig. 9i (j), Fig. 9n (k), Fig. 9p (l), Fig. 9q (m), Fig. 9s (n), Fig. 9t (o).

Supplementary Table. 1 | Accession numbers

**Gene**	**Database: Accession number**
BrSRK46	GenBank: AB013718
BrSRK8	GenBank: BAA07576
BrSRK9	GenBank: BAA06285
BrSRK12	GenBank: BAA07577
BrSRK21	GenBank: BAF57003
BrSRK29	GenBank: BAA31252
BrSRK40	GenBank: BAE78539
BrSRK44	GenBank: BAE78540
BrSRK45	GenBank: BAA34911
LaLal2	GenBank: KC981240
AhSRK13	GenBank: FO203478
BrSCR46	GenBank: AB039764
BrSCR12	GenBank: AB035503
Brassica rapa FER1 (BrFER1)	BRAD: Bra012850
Arabidopsis thaliana FER (AtFER)	BRAD: At3g51550
Brassica napus FER1 (BnFER1)	BRAD: ZS11C07G048370
Brassica napus FER2 (BnFER2)	BRAD: ZS11A03G043360
Brassica napus FER3 (BnFER3)	BRAD: ZS11A01G022250
Brassica napus FER4 (BnFER4)	BRAD: ZS11C01G028570
Brassica oleracea FER1 (BoFER1)	BRAD: BolC7t45154H
Brassica oleracea FER2 (BoFER2)	BRAD: BolC1t02833H
Camelina sativa FER1 (CsFER1)	BRAD: Csa04g037340
Camelina sativa FER2 (CsFER2)	BRAD: Csa06g025680
Camelina sativa FER3 (CsFER3)	BRAD: Csa09g058580
Raphanus sativus FER1 (RsFER1)	BRAD: Rsa10032989

Raphanus sativus FER2 (RsFER2)	BRAD: Rsa10018743
Raphanus sativus FER3 (RsFER3)	BRAD: Rsa10018058
Arabidopsis halleri FER (AhFER)	BRAD: Araha.27768s0001
Arabidopsis lyrata FER (AlFER)	BRAD: AL5G31990
BrANJ1	BRAD: Bra002541
BrANJ2	BRAD: Bra033827
BrANJ3	BRAD: Bra033828
AtPCP-Bα	EnsemblPlants: At5g61605
AtPCP-Bβ	EnsemblPlants: At2g29790
AtPCP-Bγ	EnsemblPlants: At2g16535
AtPCP-Bδ	EnsemblPlants: At2g16505
BrapaB1	Phytozome: Brara.B03493
BrapaB3(BrPCP-B3)	Phytozome: Brara.C00902
BrapaB4	Phytozome: Brara.D00928
BoPCP-B1	EnsemblPlants: Bo3g158640
BoPCP-B2	EnsemblPlants: Bo4g128470
BoB1	EnsemblPlants: Bo3g037740
BoB2	EnsemblPlants: Bo3g037750
BoB3	EnsemblPlants: Bo3g039390
BoB5	EnsemblPlants: Bo4g128460
BoB7	EnsemblPlants: Bo4g137580
BoB9	EnsemblPlants: Bo7g054270
BoB10	EnsemblPlants: Bo9g014670
BoB11	EnsemblPlants: Bo9g120820
LaPCP-B	Phytozome: Luann.0081s0081
BrROP2	BRAD: Bra016477
AtROP2	BRAD: At1g20090
BrMLPK	BRAD: Bra035659
BrARC1	BRAD: Bra010834
AtRBOHD	BRAD: At5g47910
BrRBOHA1	BRAD: Bra009266
BrRBOHA2	BRAD: Bra011911
BrRBOHB	BRAD: Bra031658
BrRBOHC1	BRAD: Bra029194
BrRBOHC2	BRAD: Bra013862

BrRBOHD1	BRAD: Bra037520
BrRBOHD2	BRAD: Bra020724
BrRBOHE1	BRAD: Bra031070
BrRBOHE2	BRAD: Bra025721
BrRBOHF	BRAD: Bra027764
BrRBOHG1	BRAD: Bra019191
BrRBOHG2	BRAD: Bra019189
BrRBOHI	BRAD: Bra033151
OsRBOHD	EnsemblPlants: Os05g0465800
BrACTIN2	BRAD: Bra037560

Supplementary Table. 2 | S or AS-ODN sequences

**S or AS-ODN**	**sequence**
S-BrSRK46	TCAGATTGGCTGCCGCTGATCT
AS-BrSRK46	AGATCAGCGGCAGCCAATCTGA
S-BrFER1	TATGATTACATGGCTCATGGGA
AS-BrFER1	TCCCATGAGCCATGTAATCATA
S-BrANJ1	GGAGCTTTAACGAGTTGTCA
AS-BrANJ1	TGACAACTCGTTAAAGCTCC
S-BrRBOHF	ACCAGCACAAGACTATAGAA
AS-BrRBOHF	TTCTATAGTCTTGTGCTGGT
S-BrARC1	AGCTCCATCGCTAGGTGGAT
AS-BrARC1	ATCCACCTAGCGATGGAGCT
S-BrMLPK	AAGCTGAGAGTCCAAGTAA
AS-BrMLPK	GTTACTTGGACTCTCAGCTT

Supplementary Table. 3 | Primers for qRT-PCR

BrACTIN2	qBrACTIN2-F	ATCCAGGCTGTTCTCTCCCT
	qBrACTIN2-R	ATCTCCCCCTCGAAACCCTC
BrSRK46	qBrSRK46-F	AAAAGGAGGACACGGCTGAG
	qBrSRK46-R	TTCACACCCATACTGCGGTC
BrSRK$^{\triangle TM}$	qBrSRK$^{\triangle TM}$-F	TGGCAAAGTTTCGATTACCCT
	qBrSRK$^{\triangle TM}$-R	TTTTGAGGTCGTAACCCAGT
BrFER1	qBrFER1-F	CTCCTCCATCGCTTATGCCTT
	qBrFER1-R	CGACCGCAGTAGAAGATCCC
BrANJ1	qBrANJ1-F	ACACCTGATAACGACACTCTTT
	qBrANJ1-R	GCTTGGAATCTTAGACACGTTC
BrRBOHF	qBrRBOHF-F	GTCTTGCTTGTGCTTCACGG
	qBrRBOHF-R	AGCGTCAGAACGTTACCAGG
AhSRK13	qAhSRK13-F	TCGTTCTGCATTACCCATCA
	qAhSRK13-R	CAAATCCCGAGATACCAACG
AtFER	qAtFER-F	CTATCGAAGACTGGTCCTACAC
	qAtFER-R	ATAGAACAACGCCAAAGGAGTA
BrMLPK	qBrMLPK -F	CGGTTGTGTCTTTAAAGGATGG
	qBrMLPK -R	AAGACGTTGCTCATCCTCTAAA
BrARC1	qBrARC1 -F	TGAAGAATTTGATCGTGCAGTG
	qBrARC1 -R	CTCTATTCTCCTTCCCGGTTTT

Supplementary Table. 4 | Primers for constructs used in Yeast two hybrid, BiFC, Pull-down, Co-IP or ROS test in infiltrated tobacoo leaves

Construct	Primer name	Sequence (5' to 3')
BrSRK46 (KD)-BD	BrSRK46(KD)-BD-F	tatggccatggaggccgaattcAGTTTGATTGTTGGAGTTAGTGTTGTT
	BrSRK46(KD)-BD-R	gtcgacggatccccgggaattcCCGGGCATCGATGACTGA
BrFER1 (KD)-AD	BrFER1(KD)-AD-F	ggccatggaggccagtgaattcGCTTACCGTAGACGTAAGGCTGG
	BrFER1(KD)-AD-R	gatgcccacccgggtggaattcCTAACGTCCCTTAGGATTCATGATC
BrFER1 (KD)-nYFP	BrFER1(KD)-nYFP-F	aaaaagcaggcttcTACGCCTCGTCCCTTCCTTC
	BrFER1(KD)-nYFP-R	agaaagctgggtcGAACTCTAAGTTCCAGAGAACATCTCC
BrSRK46 (KD)-cYFP	BrSRK46(KD)-cYFP-F	aaaaagcaggcttcGAAAATTTCTCCAATTGTAACGAACT
	BrSRK46(KD)-cYFP-R	agaaagctgggtcTGTTGCTTCACTTCCAAGCATC
GST-BrSCR12	GST-BrSCR12-F	ctccaaaatcggatctggttccgcgtggatccATGAAATCTGCAATTTATGCTTTATTATG
	GST-BrSCR12-R	gaggcagatcgtcagtcagtcacgatgcggccgcTTAGCATTTACATGTACAAAGCCGTC
GST-BrSCR46	GST-BrSCR46-F	gatctggttccgcgtggatccATGAATTCTGCTGTTTATGCTTTATTATG
	GST-BrSCR46-R	tcagtcagtcacgatgcggccgcCTATTTACAATCGCAAGAATAAGTATTCTT
BrSRK46-HA	BrSRK46-HA-F	acgggggacgagctcggtaccATGAAAGGTGTACGAAACATC
	BrSRK46-HA-R	tcgtcgactctagaggatccTTAAGCGTAATCTGGAACATCGTATGGGTACAT
MBP-BrFER1(KD)	MBP-BrFER1(KD)-F	ggaaggatttcagaattcggatccTACGCCTCGTCCCTTCCTTC
	MBP-BrFER1(KD)-R	agcatcgaattcctgcaggtcgacGAACTCTAAGTTCCAGAGAACATCTCC
BrFER1-MYC	BrFER1-MYC-F	gaagaagatcttccaatactaATGAAGATAACTGAGGGACGATCAC

	BrFER1-MYC-R	tcgtagtggatccccaatactCTGAAGCTGCAACGCGAAC
BrRBOHD2-GFP	BrRBOHD2-GFP-F	agaacacgggggactctagaATGAGACGAGGCAGTTCAGGTAA
	BrRBOHD2-GFP-R	gcccttgctcaccattctagaAAAGTTCTCTTTGTGGAAGTCAAACTT
GST-BrROP2	GST-BrROP2-F	gatctggttccgcgtggatccATGAGCGCGTCAAGGTTCAT
	GST-BrROP2-R	tcagtcagtcacgatgcggccgcTCACAAGAAGGCGCAACGG
MBP-AtROP2	MBP-AtROP2-F	ggaaggatttcagaattcggatccATGGCGTCAAGGTTTATAAAGTGTG
	MBP-AtROP2-R	agcatcgaattcctgcaggtcgacTCACAAGAACGCGCAACGG
BrSRK46(KD)-FLAG	BrSRK46(KD)-FLAG-F	ccgttgcgcaagcttctcgagaattc AGTTTGATTGTTGGAGTTAGTGTTGTT
	BrSRK46(KD)-FLAG-R	gtcgtcatcgtccttgtagtcgac CCGGGCATCGATGACTGA
BrFER1(KD, C730W)	BrFER1(KD,C730W)-F	GTGGGCACCATACTGGTACAAGAAAG
	BrFER1(KD,C730W)-R	CCAGTATGGTGCCCACTCAGCTAAGC
AtFER(ED)-FLAG	AtFER(ED)-FLAG-F	ccgttgcgcaagcttctcgagaattcGATTACTCTCCAACAGAGAAAATCCTAT
	AtFER(ED)-FLAG-R	gtcgtcatcgtccttgtagtcgacCGTATTGCTTTTCGATTTCCTAGTAG
GST-BrPCP-B3	GST-BrPCP-B3-F	gatctggttccgcgtggatccATGTCATCATCACAGTTCACTATCTTTT
	GST-BrPCP-B3-R	tcagtcagtcacgatgcggccgcTTATGGCTTGCCGCACCTA
MBP-AtPCP-Bγ	MBP-AtPCP-Bγ-F	ggaaggatttcagaattcggatccGAAAATGGAAAAAGTGTTGAAGCG
	MBP-AtPCP-Bγ-R	agcatcgaattcctgcaggtcgacATCTTTGCAGTCAGCAACACATTC
GST-BrRBOHD1(CT)	GST-BrRBOHD1(CT)-F	gatctggttccgcgtggatccGACATCATCAACAACATGAAAACTGA
	GST-BrRBOHD1(CT)-R	tcagtcagtcacgatgcggccgcTTAGAAGTTCTCTTTGTGGAAGTCAAA
GST-BrRBOHD2(CT)	GST-BrRBOHD2(CT)-F	gatctggttccgcgtggatccATCAAACCGGTTAAGATTGTCAAGG

	GST-BrRBOHD2(CT)-R	tcagtcagtcacgatgcggccgcCAAAGCTAGATTCTTTAGGTCCTTTG
GST-BrRBOHF(CT)	GST-BrRBOHF(CT)-F	gatctggttccgcgtggatccGATTTGCTTAACAACATTGTTAAAATGG
	GST-BrRBOHF(CT)-R	tcagtcagtcacgatgcggccgcTTAGAAATGCTCCTTGTGGAACTCA
MBP-BrFER1(ED)	MBP-BrFER1(ED)-F	ggaaggatttcagaattcggatccCTCCTCCTCCTTCTCATATCCTTATC
	MBP-BrFER1(ED)-R	agcatcgaattcctgcaggtcgacTGTATGGCTCTTAGATGAACCAGTG
